# Squaramate‐Modified Nucleotides and DNA for Specific Cross‐Linking with Lysine‐Containing Peptides and Proteins

**DOI:** 10.1002/anie.201906737

**Published:** 2019-08-13

**Authors:** Ivana Ivancová, Radek Pohl, Martin Hubálek, Michal Hocek

**Affiliations:** ^1^ Institute of Organic Chemistry and Biochemistry Czech Academy of Sciences Flemingovo nam. 2 16610 Prague 6 Czech Republic; ^2^ Department of Organic Chemistry Faculty of Science Charles University in Prague Hlavova 8 CZ-12843 Prague 2 Czech Republic

**Keywords:** bioconjugation, cross-linking reactions, DNA, DNA polymerase, proteins

## Abstract

Squaramate‐linked 2′‐deoxycytidine 5′‐*O*‐triphosphate was synthesized and found to be good substrate for KOD XL DNA polymerase in primer extension or PCR synthesis of modified DNA. The resulting squaramate‐linked DNA reacts with primary amines to form a stable diamide linkage. This reaction was used for bioconjugations of DNA with Cy5 and Lys‐containing peptides. Squaramate‐linked DNA formed covalent cross‐links with histone proteins. This reactive nucleotide has potential for other bioconjugations of nucleic acids with amines, peptides or proteins without need of any external reagent.

Protein–DNA interactions are of crucial importance in DNA packaging, replication, transcription, epigenetic modifications, and repair.^1^ Transcription factors (TFs) are particularly important DNA‐binding proteins that regulate gene expression through sequence‐specific binding to promoter sequences. Among the approximately 1600 known human TFs, the detailed biological role and binding motifs are fully understood only for a small fraction.^2^ Although, there is a number of methods for studying of protein–DNA interactions and for identification of DNA‐binding proteins,^3^ there is still an urgent need of other alternative methods, in particular for weakly binding proteins. Covalent cross‐linking is one of the most promising methods for identification of DNA‐binding proteins, but covalent protein–DNA conjugates are also useful for other applications in chemical biology or biosensing.^4^

There are some general non‐specific cross‐linking methods based on photochemical generation of radicals (from 5‐halouracils)^5^ or carbenes (from diazirine‐linked nucleobases)^6^ in DNA which bind randomly to neighboring amino acids through C−H activations. More challenging but potentially very useful are reactions specific for one or several amino‐acid side‐chains, but so far a very limited number of them have been reported for DNA–protein cross‐linking. Thiol‐linked DNA can cross‐link with Cys‐containing proteins through disulfide formation.^7^ Vinylsulfonamide‐linked DNA was reported to cross‐link with Cys,^8^ whereas chloroacetamide cross‐linked with proteins through Cys or His.^9^ In both cases, proximity effect was crucial for efficient formation of the covalent cross‐link between modified DNA and protein. Most frequent were reports on cross‐linking of aldehyde‐linked DNA with Lys either through inefficient and reversible Schiff‐base formation^10^ or (more often) through irreversible reductive amination,^11^, ^12^ which, requires an additional stoichiometric reductant (for example, toxic NaBH_3_CN, which complicates any in cellulo or in vivo usage). Lys–DNA interactions are very frequent and important, in particular in histones. So far, no reactive nucleobase‐modification in DNA has been reported to form irreversible cross‐links with Lys without an external reagent.

Mono‐amides of squaric acid (squaramates) are often used for bioconjugations with Lys and other amines.^13^ Diamides (squaramides) have been used as a phosphate surrogate in nucleotide^14^ or oligonucleotide (ON) analogues.^15^ A chemically synthesized 2′‐sugar‐linked squaramate–RNA conjugate, prepared through reaction of 2′‐amino‐modified RNA with diethyl squarate, was reported to cross‐link to aminoacyl‐transferase FemX_Wv_,^16^ as the only example of its use in nucleic‐acid conjugation. Within the framework of our program aimed at base‐functionalized nucleic acids for applications in chemical biology,^17^ we designed novel squaramate‐linked cytosine 2′‐deoxyribonucleoside triphosphate (dNTP) for the enzymatic synthesis of modified DNA and cross‐linking with proteins.

The synthesis of the desired modified nucleotides started with the preparation of 5‐(3‐aminopropynyl)‐2′‐deoxycytidine (**1**) by deacylation of known trifluoroacetylamide,^18^ see Scheme S1 in Supporting Information. The reaction of amine **1** with 2 equiv of diethyl squarate gave the squaramate‐linked nucleoside **dC^ESQ^** in 80 % yield (Scheme 1). Standard Yoshikawa phosphorylation^19^ with POCl_3_ gave the monophosphate **dC^ESQ^MP** in 37 % yield, whereas the triphosphorylation^20^ with POCl_3_ followed by pyrophosphate and triethylammonum bicarbonate (TEAB) gave the triphosphate **dC^ESQ^TP** in 7 % yield. The monophosphate **dC^ESQ^MP** served as model compound for reactions with Lys and peptides (Scheme 1). The reaction of **dC^ESQ^MP** with Ac‐Lys or a Lys‐containing tripeptide proceeded at room temperature in borate buffer (pH 9) overnight to give the desired conjugates **dC^ESQLys^MP** or **dC^ESQ3^** ^**pept**^ **MP** in 54 and 63 %, respectively. These nucleotide–peptide conjugates were isolated by HPLC in pure form and fully characterized by NMR spectroscopy and mass spectrometry (MS) to confirm the expected formation of the amide bond with Lys.

**Scheme 1:**
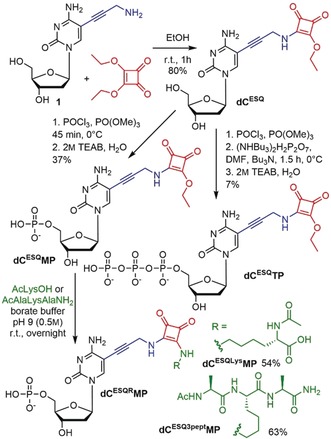
Synthesis of squaramate‐modified 2′‐deoxycytidine and 2′‐deoxycytidine mono‐ and triphosphate and **dC^ESQ^MP** adducts with Ac‐Lys and lysine‐containing tripeptide (AcAlaLysAlaNH_2_).

Then, we tested the squaramate‐linked dNTP (**dC^ESQ^TP**) as substrate for DNA‐polymerase‐catalyzed synthesis of modified DNA (Figure 1 a). First, we performed the primer extension (PEX) in the presence of KOD XL DNA polymerase with either 19‐, 20‐, 31‐ or 98‐mer template and a 13‐, 15‐ or 25‐mer primer (for the oligonucleotide sequences, see Tables S1 and S2 in the Supporting Information). In all cases (Figure 1 b and Supporting Information, Figure S1), we observed the formation of full‐length PEX products containing one, four or eighteen **dC^ESQ^** modifications. PCR amplification with either 98‐bp or 235‐bp templates also proceeded well, giving a strong band corresponding to the modified amplicon (Supporting Information, Figure S2). This shows that the reactive **dC^ESQ^TP** nucleotide does not react with DNA polymerase (neither during extension nor when modified DNA is used as template) and is a good substrate and building block for the enzymatic synthesis of reactive, modified DNA probes.

**Figure 1:**
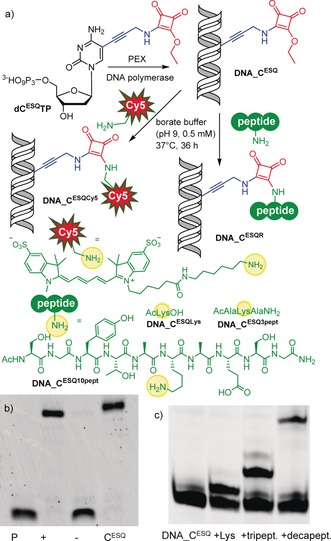
a) Synthesis of squaramate‐modified DNA (**DNA_C^ESQ^**) and its cross‐linking with Sulfo‐Cy5‐NH_2_, lysine, tri‐ and decapeptide. b) SDS‐PAGE analysis of the incorporation of **dC^ESQ^TP** into DNA using KOD XL polymerase and temp^20 1C^. P: primer^A^; (+): natural dNTPs; (−): natural dNTPs without dCTP; C^ESQ^: **dC^ESQ^TP**, dGTP, dTTP, dATP. c) SDS‐PAGE analysis of the conjugation of **DNA_C^ESQ^** (4.3 μm) with Ac‐Lys (11 mm), tripeptide (11 mm), and decapeptide (11 mm). Conditions: borate buffer (0.5 m, pH 9), 37 °C, 36 h. For the oligonucleotide sequence see Table S1 in the Supporting Information.

Next, we tested the cross‐linking reactions of **dC^ESQ^**‐linked DNA with amines and peptides (Figure 1 a,c). The reactions of 20‐bp **DNA_C^ESQ^** were performed at room temperature in borate buffer (pH 9). The reaction with Sulfo‐Cy5‐NH_2_ (100 equiv) gave the desired fluorescently labeled **DNA_C^ESQCy5^** (Supporting Information, Figure S3). Analogous reactions of **DNA_C^ESQ^** with Ac‐Lys and Lys‐containing tripeptide or decapeptide were conducted with large excess (approximately 2500 equiv) of the peptides, since no proximity effect was expected in these non‐DNA‐binding peptides. Under these conditions, the reactions proceeded with moderate efficiency and gave the desired cross‐linked conjugates **DNA_C^ESQLys^**, **DNA_C^ESQ3pept^** or **DNA_C^ESQ10pept^** with 50, 43, and 20 % conversions, respectively (Figure 1 c). All conjugates were characterized and confirmed by MALDI MS (Supporting Information, Table S4).

Finally, the reactive DNA probe **DNA_C^ESQ^** was tested in reactions with proteins. We used bovine serum albumin (BSA) as negative control of a protein containing 60 lysines that does not interact with DNA, the core‐domain of p53^21^ as a DNA‐binding protein containing lysines but not in the binding site, and a set of recombinant H2A, H2B, H3.1, and H4 histones, as examples of Lys‐rich proteins that strongly bind to DNA. The cross‐linking reactions were performed with only 2 equiv of the corresponding proteins. Since the histones are known to form dimers and oligomers, we assume that this ratio is probably effectively close to equimolar. To be closer to physiological conditions, we used phosphate (or TRIS or HEPES, see Figure S8 in the Supporting Information) buffers (pH 7.4).

At first, we performed a simple kinetic study of the cross‐linking reaction of **DNA_C^ESQ^** with histone H3.1 to show that the reaction reaches the maximum conversion in 16–24 h (Supporting Information, Figure S5). Therefore, we used 36 h reactions in other cases to ensure sufficient conversions. Figure 2 shows the results of the cross‐linking experiments with proteins. To our delight, the reactions of **DNA_C^ESQ^** with all four recombinant histones gave the covalent cross‐linked conjugates with lower mobility on a denaturing SDS‐PAGE gel (Figure 2 b). The conversions of these reactions calculated from the SDS‐PAGE were 31–34 % (Supporting Information, Table S5). The identity of the covalent DNA–protein conjugates with H2B, H3.1, and H4 histones was also confirmed by SDS‐PAGE with protein staining (Coomassie Blue, Supporting Information, Figure S7) and by HPLC‐MS analysis using electrospray ionization (Supporting Information, Figures S16–S18). Also, a longer 98‐bp PEX product containing 18 squaramate groups reacted with histone H3.1, though mixture of cross‐linked products was obtained (Supporting Information, Figure S10). On the other hand, **DNA_C^ESQ^** did not cross‐link with BSA or p53 (Supporting Information, Figure S9) or with DNA polymerase during the PEX or PCR. These results show that the proximity effect (presence of lysine(s) close to the DNA‐binding site of protein) is crucial for efficient cross‐linking in the absence of large excess of the peptide or protein.

**Figure 2:**
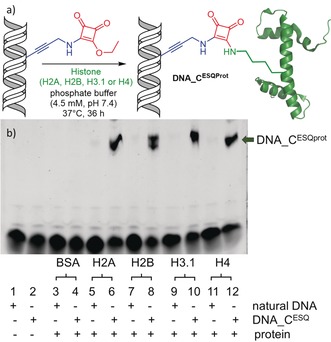
a) Cross‐linking of squaramate‐modified DNA (**DNA_C^ESQ^**) with histone recombinant proteins. b) SDS‐PAGE analysis of cross‐linking experiments with natural or modified DNA and various recombinant proteins (2 equiv of protein to DNA): 17.5 % SDS‐PAGE gel. Conditions: phosphate buffer (4.5 mm, pH 7.4), 37 °C, 36 h.

In conclusion, we designed and synthesized a novel squaramate‐linked dNTP (**dC^ESQ^TP**) and demonstrated that it was a very good substrate for KOD XL DNA polymerase in PEX or PCR synthesis of reactive DNA probes. The squaramate group reacts with amines to form a stable covalent diamide (squaramide) linkage. We have shown that the **dC^ESQ^**‐modified DNA probes reacted with amino‐linked Cy5 to form fluorescently labeled DNA. Its reactions with Lys‐containing peptides proceeded only when a large excess of the peptide was present. On the other hand, in reactions with Lys‐containing DNA‐binding proteins, where the proximity effect helps, the reactions proceed with good conversions even in almost equimolar ratio. Compared to previously reported DNA–Lys conjugations based on reductive amination,^11^, ^12^ the squaramate modification and its transformation to a stable amide proceeds under physiological conditions (at pH 7.4–9) and does not require any external reagent (i.e. toxic NaBH_3_CN used in reductive aminations^11^, ^12^). Therefore, this reactive modification and the presented methodology has good potential in the post‐synthetic labeling of DNA,^22^ bioconjugations of DNA with peptides, proteins or other biomolecules,^4^ as well as in cross‐linking experiments to identify and study DNA‐binding proteins. Further research along these lines is under way in our lab.

## Conflict of interest

The authors declare no conflict of interest.

## Supporting information

As a service to our authors and readers, this journal provides supporting information supplied by the authors. Such materials are peer reviewed and may be re‐organized for online delivery, but are not copy‐edited or typeset. Technical support issues arising from supporting information (other than missing files) should be addressed to the authors.

Supplementary
