# Supplementary material for: Squaramate‐Modified Nucleotides and DNA for Specific Cross‐Linking with Lysine‐Containing Peptides and Proteins
Source: Angew Chem Int Ed Engl. 2019 Aug 13;58(38):13345–8. doi: 10.1002/anie.201906737 (PMC6771961; doi:10.1002/anie.201906737)
Supplement: Supplementary file 1 — Supplementary [file ANIE-58-13345-s001.pdf]

Angewandte Chemie
Eine Zeitschrift der Gesellschaft Deutscher Chemiker

Supporting Information

Squaramate-Modified Nucleotides and DNA for Specific Cross-Linking with Lysine-Containing Peptides and Proteins

*Ivana Ivancová, Radek Pohl, Martin Hubálek, and Michal Hocek**

anie_201906737_sm_miscellaneous_information.pdf

Supporting information

Contents

1. Additional results, schemes, tables and figures	S2
2. Experimental section	S9
3. Copies of MALDI-TOF mass spectra	S23
4. Copies of MS spectra of DNA-protein cross-links	S27
5. Copies of NMR spectra	S30
6. References	S40

1. Additional results, schemes, tables and figures

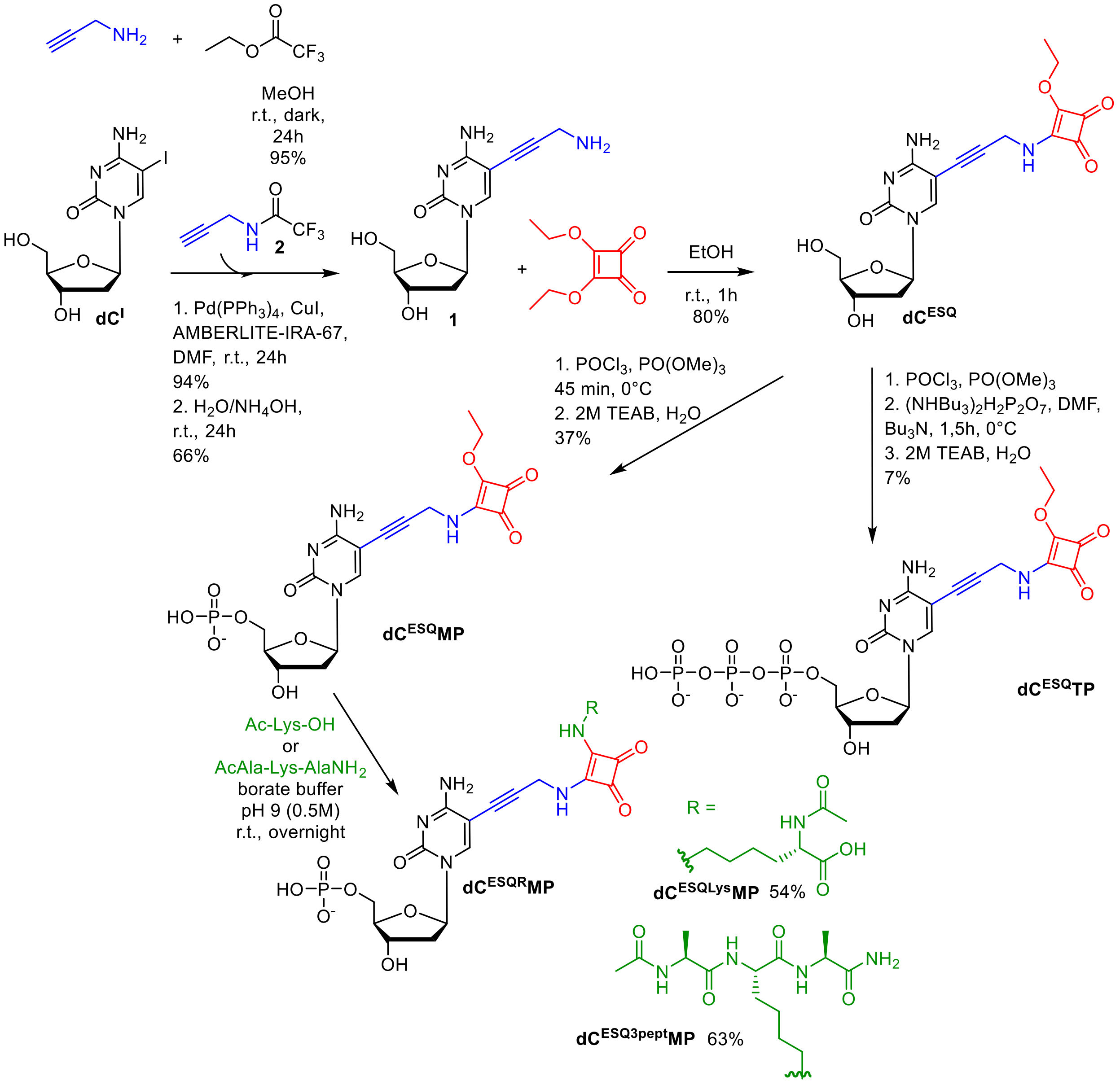

Scheme S1. Chemical synthesis of ESQ modified 2'-deoxycytidine and 2'-deoxycytidine mono- and triphosphate and 2'-deoxycytidine monophosphate adducts with Ac-Lys-OH and lysine containing tripeptide (Ac-Ala-Lys-Ala-NH_2).

Table S1. List of oligonucleotides synthesized by PEX and used in this study.[a]

Oligonucleotide	Sequence (5′→3′)	Lenght
prim^{A}	5′-AGACATGCCTAGA-3′	13-mer
prim^{B}	5′-CATGGGCGGCATGGG-3′	15-mer
prim$^{REV_LT25-TH}$	5′- CAAGGACAAAATACCTGTATTCCTT-3′	25-mer
temp^{19_1C}	5′-CCCGCCCATGCCGCCCATG-3′	19-mer
temp^{20_1C}	5′-AAACATGTCTAGGCATGTCT-3′	20-mer
temp^{31_4C}	5′-CTAGCATGAGCTCAGTCCCATGCCGCCCATG-3′	31-mer
temp^{FVL-A}	5′-GACATCATGAGAGACATCGCCTCTGGGCTAAT<br>AGGACTACTTCTAATCTGTAAGAGCAGATCCCTGG<br>ACAGGCAAGGAATACAGGTATTTTGTCCTTG-3′	98-mer
ON_C^{ESQ_19}	5′-CATGGGCGGCATGGG**C**GGG-3′	19-mer
ON_C^{ESQ}	5′-AGACATGCCTAGA**C**ATGTTT-3′	20-mer
ON_4C^{ESQ}	5′-CATGGGCGGCATGGGA**C**TGAG**C**T**C**ATG**C**TAG -3′	31-mer
DNA_C^{ESQ_19}	3′-GTACCCGCCGTACCCGCCC-5′<br>5′-CATGGGCGGCATGGG**C**GGG-3′	19-mer
DNA_C^{ESQ}	3′-TCTGTACGGATCTGTACAAA-5′<br>5′-AGACATGCCTAGA**C**ATGTTT-3′	20-mer
DNA_4C^{ESQ}	3′-GTACCCGCCGTACCCTGACTCGAGTACGATC-5′<br>5′-CATGGGCGGCATGGGA**C**TGAG**C**T**C**ATG**C**TAG -3′	31-mer
DNA_C^{ESQ_98}	3′-GTTCCTGTTTTATGGACATAAGGAACGGACAGG<br>TCCCTAGACGAGAATGTCTAATCTTCATCAGGATA<br>ATCGGGTCTCCGCTACAGAGAGTACTACAG-5′<br>5′-CAAGGACAAAATACCTGTATTCCTTG**CC**TGT**CC**<br>AGGGAT**C**TG**C**T**C**TTA**C**AGATTAGAAGTAGT**CC**TAT<br>TAG**CCC**AGAGG**C**GATGT**C**T**C**T**C**ATGATGT**C**-3′	98-mer

[a] primers are underlined, nucleotides bearing modification are in bold

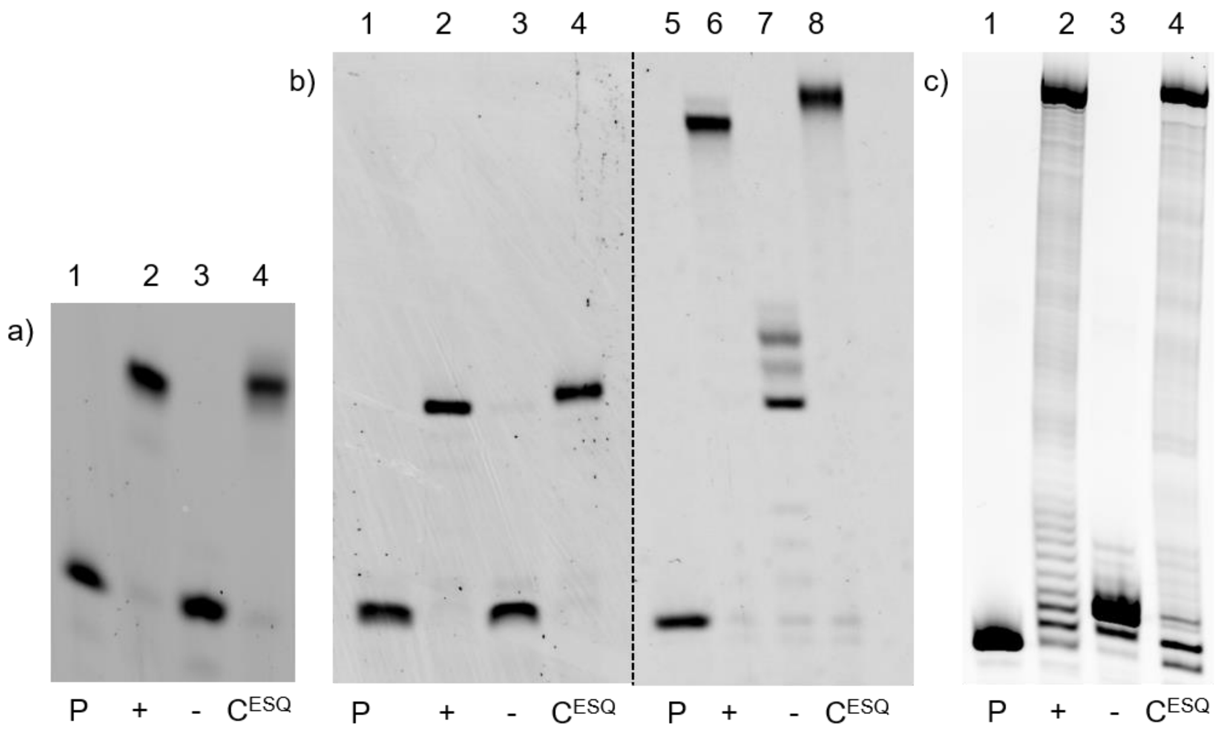

Figure S1. Primer extension using KOD XL polymerase and temp^{19_1C} (a; lines 2-4), temp^{20_1C} (b; lines 2-4), temp^{31_4C} (b; lanes 6-8) and temp$^{FVL\text{-}A}$ (c; lanes 2-4). a) Lane 1 (P): primer^{B}; lane 2 (+): natural dNTPs; lane 3 (-): negative control without dCTP; lane 4 (C^{ESQ}): **dC^{ESQ}TP**, dGTP. b) Lane 1 (P): primer^{A}; lane 5 (P): primer^{B}; lanes 2,6 (+): natural dNTPs; lanes 3,7 (-): negative control without dCTP; lanes 4,8 (C^{ESQ}): **dC^{ESQ}TP**, dGTP, dTTP, dATP. c) Lane 1 (P): prim$^{REV_LT25\text{-}TH}$; lane 2 (+): natural dNTPs; lane 3 (-): negative control without dCTP; lane 4 (C^{ESQ}): **dC^{ESQ}TP**, dGTP, dTTP, dATP.

Table S2. List of oligonucleotides used in PCR experiments.

Oligonucleotide	Sequence (5′→3′)	Lenght
prim^{FOR_L20}	5′- GACATCATGAGAGACATCGC-3′	20-mer
prim$^{REV_LT25\text{-}TH}$	5′- CAAGGACAAAATACCTGTATTCCTT-3′	25-mer
prim^{FOR_235}	5′-CGTCTTCAAGAATTCTATTTGACA-3′	24-mer
prim^{REV_235}	5′-GGAGAGCGTTCACCGACA-3′	18-mer
temp$^{FVL\text{-}A}$	5′-GACATCATGAGAGACATCGCCTCTGGGCTAATAGG ACTACTTCTAATCTGTAAGAGCAGATCCCTGGACAGGC AAGGAATACAGGTATTTTGTCCTTG-3′	98-mer
temp^{PCR_235}	5′-CGTCTTCAAGAATTCTATTTGACAAAAATGGGC TCGTGTTGTACAATAAATGTGTCTAAGCTTGGGTCCCA CCTGACCCCATGCCGAACTCAGAAGTGAAACGCCGTA GCGCCGATGGTAGTGTGGGGTCTCCCCATGCGAGAGTA GGGAACTGCCAGGCATCAAATAAAACGAAAGGCTCAG TCGAAAGACTGGGCCTTTCGTTTTATCTGTTGTTTGTCG GTGAACGCTCTCC-3′	235-mer

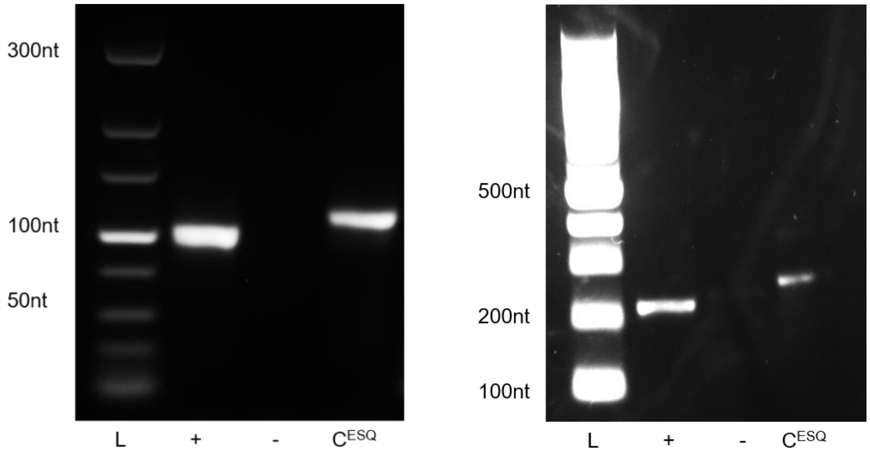

Figure S2. Agarose gel analysis of PCR product amplified by KOD XL DNA polymerase; (L): ladder; (+): natural dNTPs; (-): negative control without dCTP; (C^{ESQ}): **dC^{ESQ}TP,** dATP, dGTP, dTT; 2% (1.3%) agarose gel for 98-mer (235-mer) stained with GelRed.

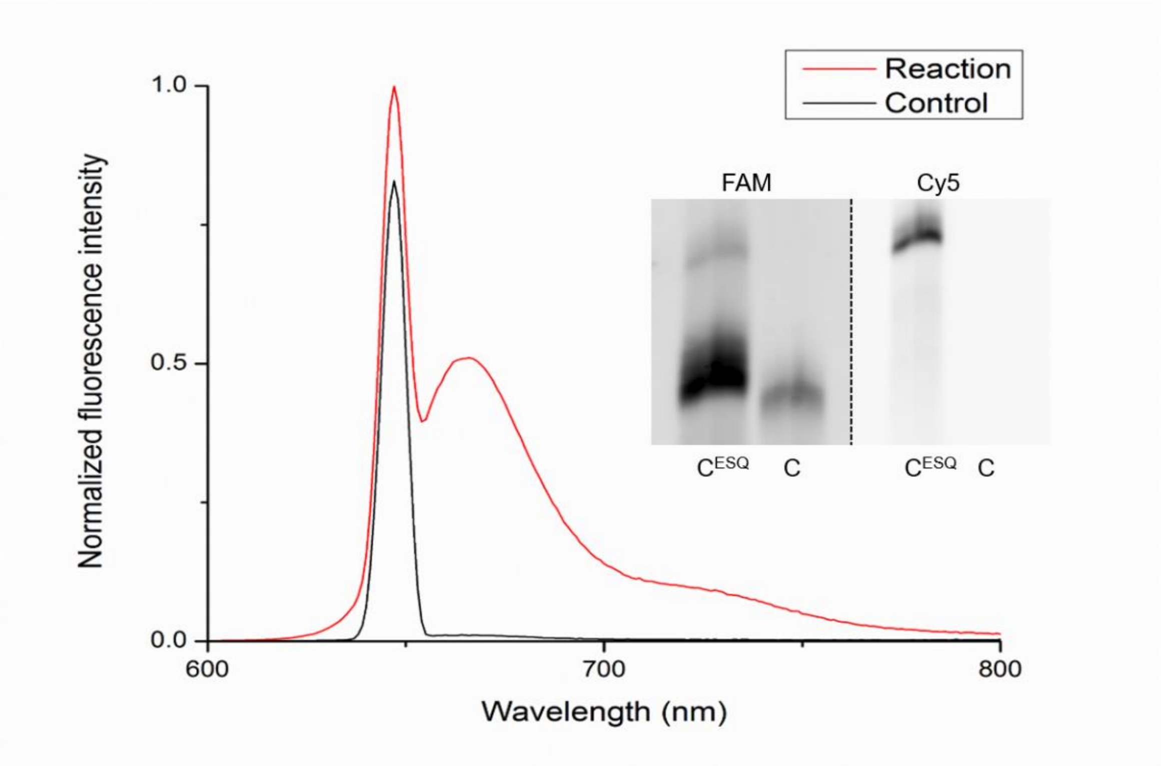

Figure S3. Conjugation of natural and ethoxy squarate modified **DNA_C^{ESQ}** with sulfo-Cy-5-amine. Conditions: FAM labeled natural DNA (Control line in black) or FAM labeled **DNA_C^{ESQ}** (Reaction line in red), borate buffer (0.5 M, pH 9), 37°C, and 36 h. Fluorescence of the products was measured on spectrofluorimeter (water; λ_{ex}=646 nm, λ_{em}=662 nm). PAGE analysis: C line: natural DNA + sulfo-Cy-5-NH_2; C^{ESQ} line: **DNA_C^{ESQ}** + sulfo-Cy-5-NH_2. Excited with 473 nm for FAM and 635 nm for Cy5.

Table S3. List of aminoacids and synthetic peptides used to test cross-linking abilities of **DNA_C^{ESQ}**.

	Peptide Sequence	Molecular weight / Da	% conversion
Acetyl-lysine		188.23	50%
tripeptide	Ac-AKA-NH_2	329.2	43%
decapeptide	Ac-SGYTAKAQSG-NH_2	1011.47	20%

Note: Conversions were determined from the gel using ImageJ Quantificator

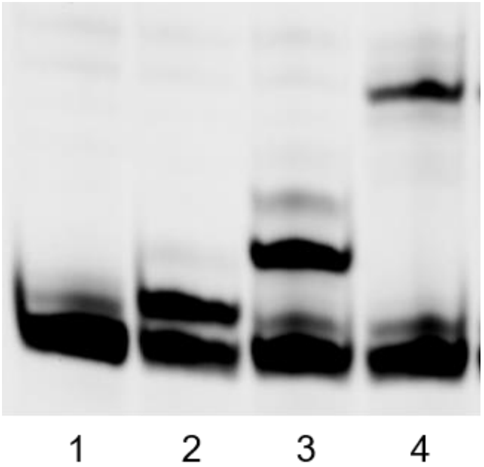

Figure S4. Conjugation of **DNA_C^{ESQ}** (4.3 µM; line 1) with Ac-lysine (11 mM; line 2), tripeptide (11 mM; lane 3) and decapeptide (11 mM; line 4). Conditions: borate buffer (0.5 M, pH 9), 37°C, 36 h.

Table S4. MALDI data of modified oligonucleotide ON_C^{ESQ_19}, ON_C^{ESQ} and adducts of DNA_C^{ESQ} with Ac-lysine, tripeptide and decapeptide (for MALDI spectrum see Figures S11-S15). Single stranded conjugates were obtained after magnetoseparation.

ON	*M* (calc.) / Da	*M* (found) $[M+H]^+$ / Da
ON_C^{ESQ_19}	6127.9	6128.1
ON_C^{ESQ}	6293.3	6294.3
ON_C^{ESQLys}	6435.5	6436.4
ON_C^{ESQ3pept}	6576.5	6576.6
ON_C^{ESQ10pept}	7257.8	7258.0

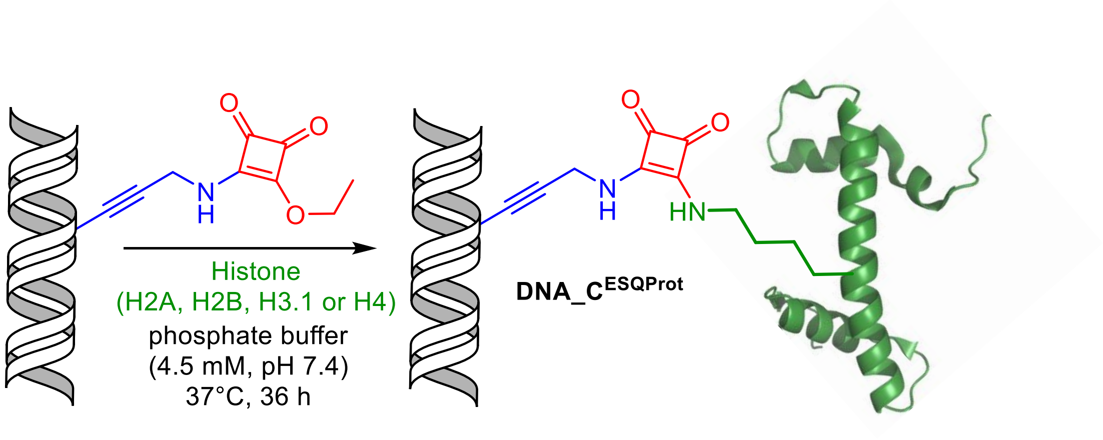

Scheme S2. Cross-linking of squaramate modified DNA (**DNA_C^{ESQ}**) with histone recombinant proteins.

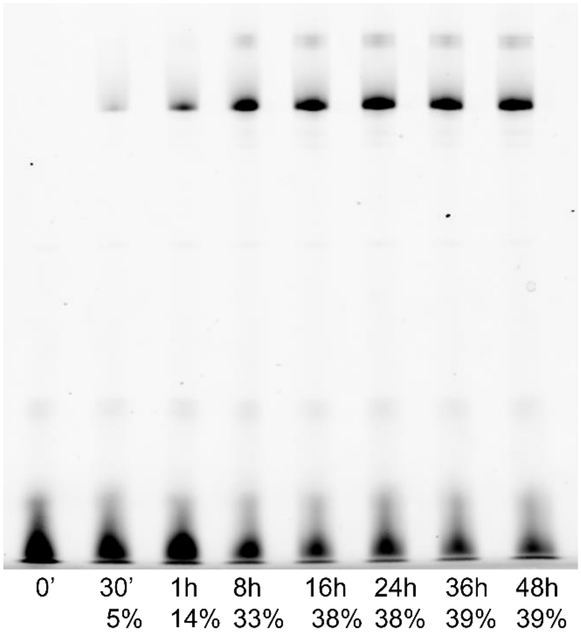

Figure S5. Effect of reaction time on cross-link formation between **DNA_C^{ESQ}** and histone H3.1: 17.5% SDS gel. Conditions: 2 equiv. of protein to DNA, phosphate buffer (4.5 mM, pH 7.4), 37°C.

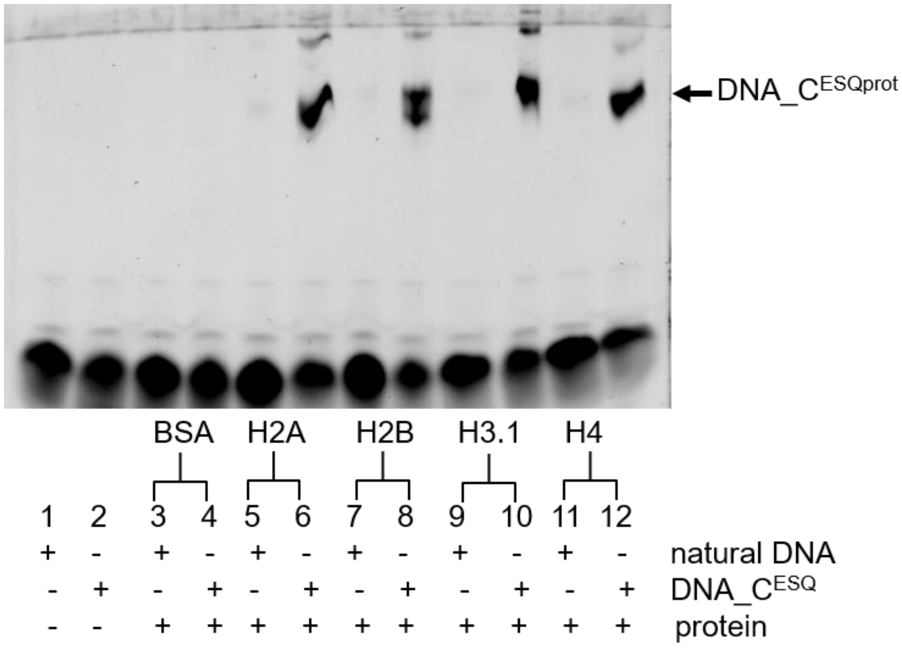

Figure S6. SDS analysis of control experiment with natural or modified DNA and various recombinant proteins (2 equiv. of protein to DNA): 17.5% SDS gel. Conditions: phosphate buffer (4.5 mM, pH 7.4), 37°C, 36 h.

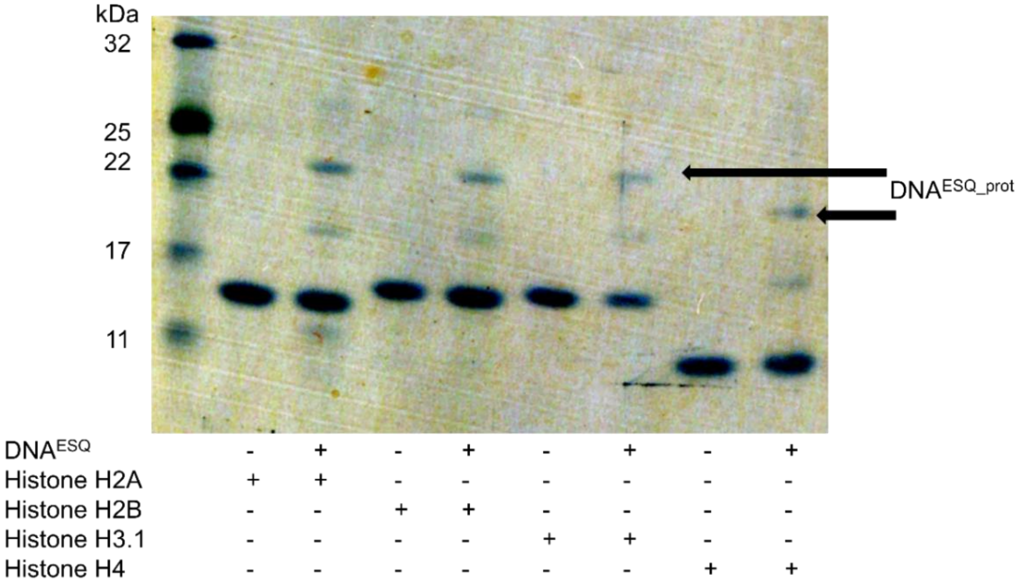

Figure S7. SDS-PAGE analysis of DNA-protein cross-link between **DNA_C^{ESQ}** and recombinant proteins followed by Coomassie staining: 17.5% SDS gel. Conditions: 3 equiv. of DNA to protein, phosphate buffer (4.5 mM, pH 7.4), 37°C, 36 h.

Table S5. DNA-protein cross-link conversions between **DNA_C^{ESQ}** and recombinant proteins.

Protein	Molecular weight / kDa	DNA binding	No. of Lys	% conversion
BSA	69.3	no	60	0 %
GST_p53_CD	78.5	yes	21	0%
H2A	13.99	yes	13	34%
H2B	13.79	yes	20	34%
H3.1	15.27	yes	13	31%
H4	11.24	yes	11	34%

Note: Conversions were determined from the gel using ImageJ Quantificator

Table S6. MS data of DNA-protein conjugates (for MS spectrum see Figures S16-S18).

	M (calc.) / Da	*M* (found) / Da
ON_C^{ESQH2B}	20036.78	19905
ON_C$^{ESQH3.1}$	21520.63	21388
ON_C^{ESQH4}	17484.11	17351

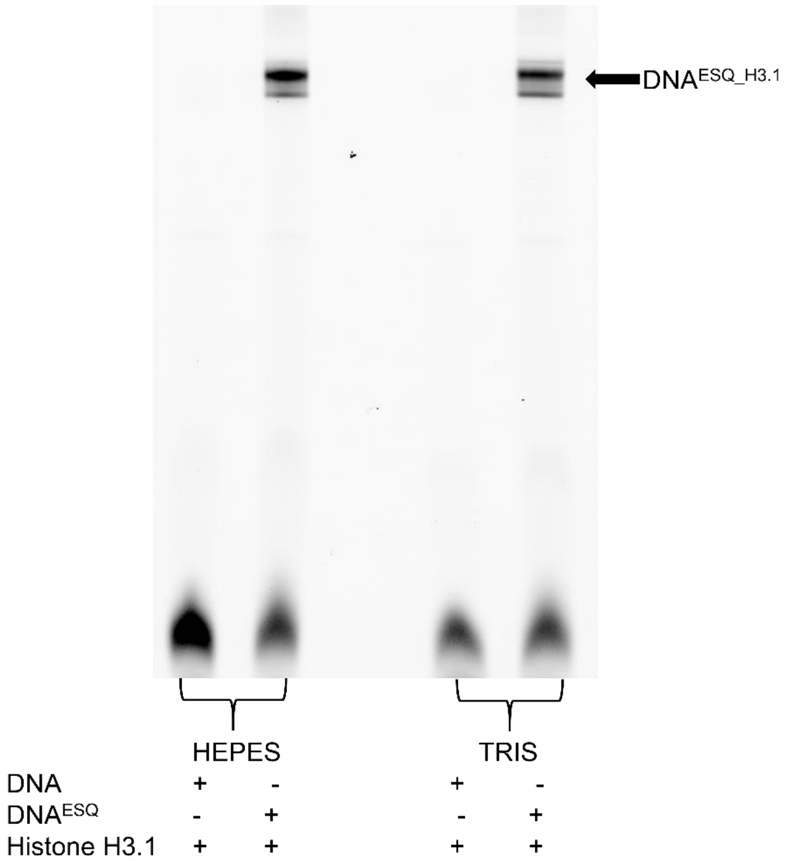

Figure S8. Reaction of the **DNA_C^{ESQ}** with histone H3.1 using different buffers. Conditions: 2 equiv. of protein to DNA, HEPES buffer (100 mM, pH 7.4) or TRIS buffer (100 mM, pH 7.4), 37°C, 36 h, 17.5% SDS gel. Conversions (determined from the gel using ImageJ Quantificator): HEPES: 34%, TRIS: 29%.

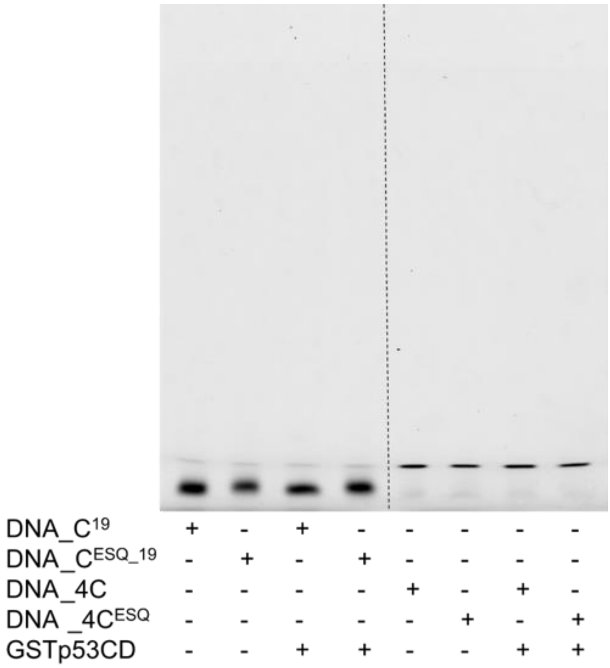

Figure S9. 10% SDS analysis of control experiment with natural or modified DNA and GST_p53_CD protein. Conditions: DNA (5 pmol), pH 7.6, 37°C, 36 h.

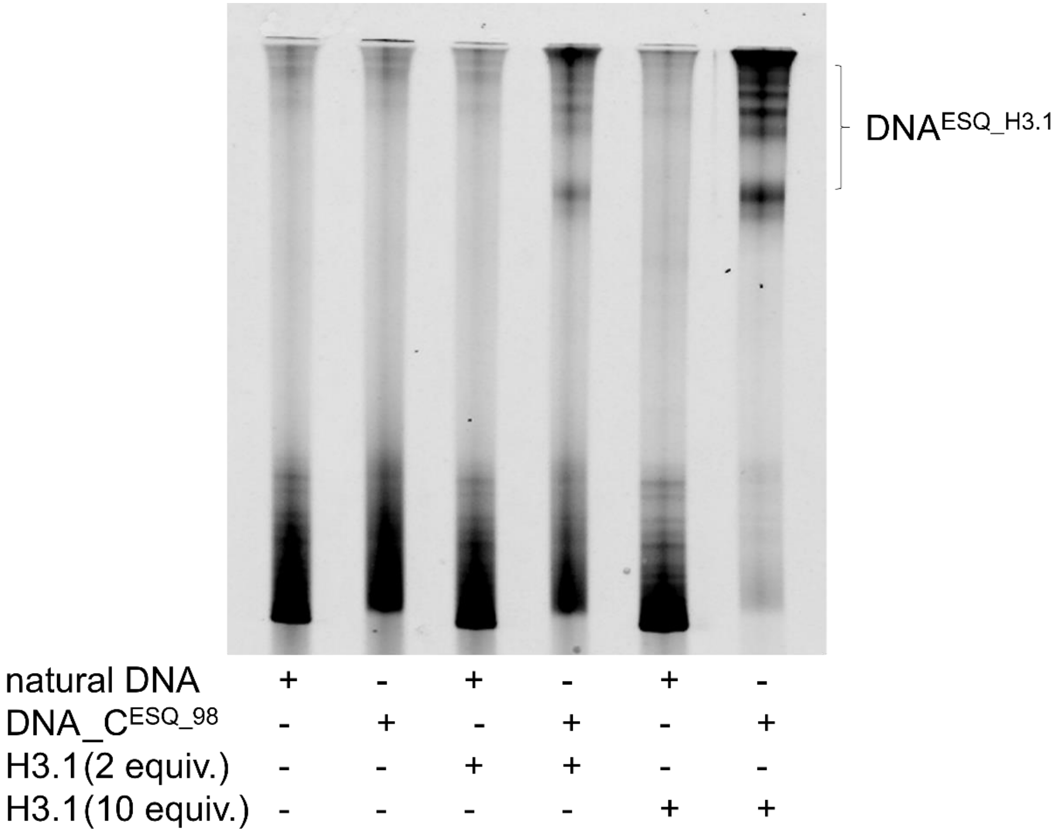

Figure S10. SDS analysis of cross-links of natural or modified 98-mer PEX product and H3.1 histone protein (2 resp. 10 equiv. of protein to DNA): 4-12% SDS gel. Conditions: phosphate buffer (4.5 mM, pH 7.4), 37°C, 36 h.

2. Experimental section

2.1 General remarks for the synthetic part

NMR spectra were recorded on a Bruker Avance-IIIHD 500 (500.0 MHz for ^{1}H, 202.4 MHz for ^{31}P, 125.7 MHz for ^{13}C) spectrometer. ^{1}H and ^{13}C resonances were assigned using H, H-COSY, H,C-HSQC and H,C-HMBC experiments. The samples were measured in D_2O, CD_3OD or DMSO-d_6. Chemical shifts (δ scale, in ppm) were referenced as follows: D_2O (referenced to dioxane as internal standard: 3.75 ppm for ^{1}H NMR and 69.30 ppm ^{13}C NMR); CD_3OD (referenced to solvent signal: 3.31 ppm for ^{1}H NMR and 49.00 ppm for ^{13}C NMR); DMSO-d_6 (referenced to solvent signal: 2.50 ppm for ^{1}H NMR and 39.70 ppm for ^{13}C NMR). ^{31}P chemical shifts were referenced to H_3PO_4 as an external standard (0 ppm). Coupling constants (*J*) are given in Hz. High resolution mass spectra were measured on LTQ Orbitrap XL (Thermo Fisher Scientific) using ESI ionization technique. All materials were purchased from commercial suppliers and used without further purification unless otherwise stated. $POCl_3$ and $PO(OMe)_3$ were distilled prior to use. The water used in synthetic part was of HPLC quality. Purification of nucleoside triphosphates was performed using HPLC (Waters) on a C18 reversed phase column (Phenomenex, Luna C18 (2) 100 Å).

2.2 Synthesis and characterization of ESQ modified 2'-deoxycytidine and 2'-deoxycytidine mono- and triphosphate and 2'-deoxycytidine monophosphate adducts with Ac-Lys-OH and lysine containing tripeptide (Ac-Ala-Lys-Ala-NH$_2$)

2, 2, 2-Trifluoro-*N*-(prop-2-ynyl)acetamide (2)[1]

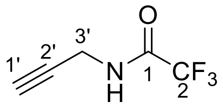

Propargylamine (1.50 g, 27.2 mmol) and ethyl trifluoroacetate (5.03 g, 35.4 mmol) were dissolved in methanol (50 mL) and stirred for 24 h in the dark at room temperature. After removal of the solvent under reduced pressure saturated $NaHCO_3$ solution (56 mL) was added to the residue and the aqueous phase was extracted with DCM (4x70 mL). The combined organic layers were dried

over Na_2SO_4, filtered and the solvent was removed under reduced pressure. Desired product was obtained as yellow oil in 95% yield (3.89 g) and used without further purification.
All spectral data are consistent with the published literature.

5-[3-(Trifluoroacetamido)-prop-1-ynyl]-2'-deoxycytidine (dC^{PACF3})[2]

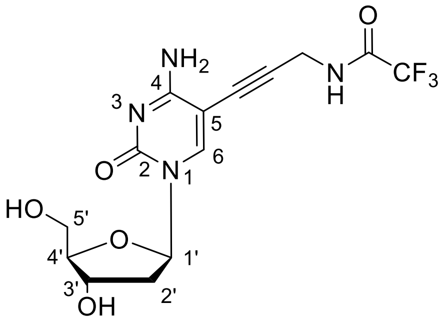

5-Iodo-2'-deoxycytidine (**dC^{I}**; 1.11 g, 3.16 mmol), *N*-propargyl trifluoroacetamide (1.44 g, 9.50 mmol), CuI (0.120 g, 0.632 mmol), $Pd(PPh_3)_4$ (0.365 g, 0.316 mmol) and Amberlite Ira-67 resin (2,81 g) were placed in a 50 mL round bottom flask and dissolved in anhydrous DMF (17 mL) under argon atmosphere. The reaction mixture was stirred in the dark at r.t. for 24 hours then filtered over bed of SiO_2 topped with celite. The solvents were removed under reduced pressure and the title product was obtained by column chromatography using DCM/methanol (7:1) as a mobile phase in 94% yield (1.12 g) as an orange oil.
All spectral data are consistent with the published literature.

5-(3-Amino-1-propynyl)-2'-deoxycytidine (1)

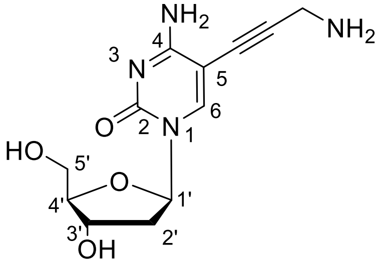

dC^{PACF3} (1.28 g, 3.40 mmol) was dissolved in 17 mL of HPLC water in the pressure tube. After complete dissolution, aqueous ammonium hydroxide (92 mL) was added and the tube was closed properly. The reaction was stirred 24 hours at r.t. and then concentrated down. The crude product was redissolved in HPLC H_2O (26 mL) and DOWEX 50x8 resin (7.00 g) was added. The mixture was stirred for 30 min and filtered over a bed of DOWEX 50x8 resin (7.00 g) which was then

washed with HPLC H_2O and the product was eluted off the resin with HPLC H_2O/conc. NH_4OH (4:1, 800 mL). Final product was obtained as orange-brown oil (0.627 g, 66%) after removal of the solvents under reduced pressure.

^{1}H NMR (500.0 MHz, CD_3OD): 2.12 (ddd, 1H, J_{gem} = 13.4, $J_{2'b,1'}$ = 6.7, $J_{2',3'}$ = 6.2, H-2′b); 2.38 (ddd, 1H, J_{gem} = 13.4, $J_{2'a,1'}$ = 6.1, $J_{2'a,3'}$ = 4.0, H-2′a); 3.61 (s, 2H, CH_2N); 3.73 (dd, 1H, J_{gem} = 12.1, $J_{5'b,4'}$ = 3.6, H-5′b); 3.81 (dd, 1H, J_{gem} = 12.1, $J_{5'a,4'}$ = 3.1, H-5′a); 3.95 (ddd, 1H, $J_{4',3'}$ = 4.0, $J_{4',5'}$ = 3.6, 3.1, H-4′); 4.36 (dt, 1H, $J_{3',2'}$ = 6.2, 4.0, $J_{3',4'}$ = 4.0, H-3′); 6.21 (dd, 1H, $J_{1',2'}$ = 6.7, 6.1, H-1′); 8.30 (s, 1H, H-6). ^{13}C NMR (125.7 MHz, CD_3OD): 32.06 (CH_2N); 42.38 (CH_2-2′); 62.49 (CH_2-5′); 71.78 (CH-3′); 74.48 (cyt-**C**≡C-CH_2); 87.90 (CH-1′); 89.05 (CH-4′); 92.70 (C-5); 95.95 (cyt-C≡**C**-CH_2); 145.46 (CH-6); 156.77 (C-2); 166.49 (C-4).

HR/MS (ESI$^-$) for $C_{12}H_{15}O_4N_4$: [M - H]$^-$ calculated 279.10988, found 279.10999.

3-[5- (3-Amino-1-propynyl) -2'-deoxycytidine]-4- ethoxycyclobut-3-ene-1,2-dione (dC^{ESQ})

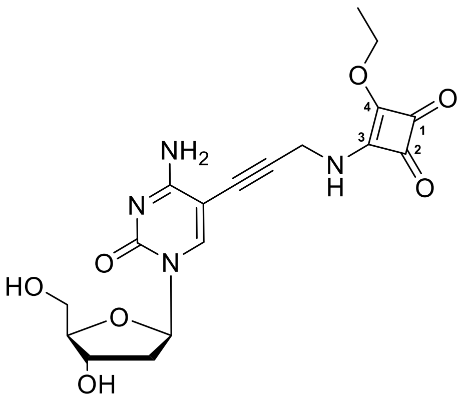

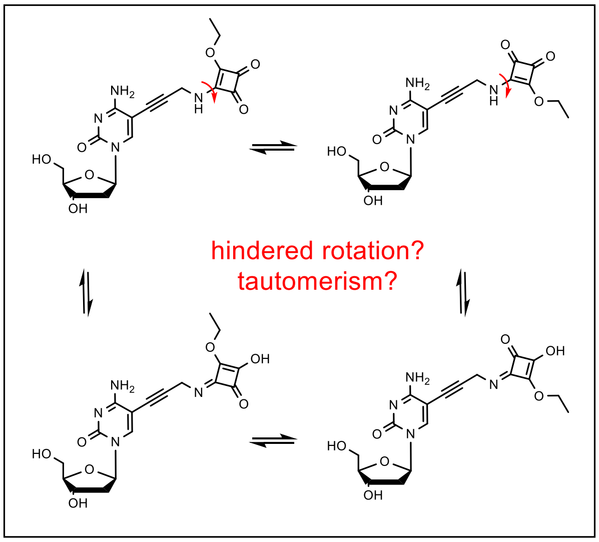

5 (0.200 g, 0.713 mmol) was well suspended in 4 ml of ethanol. Diethyl squarate (0.211 mL, 1.43 mmol) was added dropwise during 30 min to the stirred solution and the reaction mixture was stirred another 1 hour at room temperature. Column chromatography using DCM/methanol (8:2) as a mobile phase gave the desired product as white powder in 80% yield (0.231g).

^{1}H NMR (500.0 MHz, DMSO-d_6, T = 100 °C): 1.40 (t, 3H, J_{vic} = 7.0, C**H**$_3$$CH_2O$); 2.00 (dt, 1H, J_{gem} = 13.4, $J_{2'b,1'}$ = 7.0, $J_{2',3'}$ = 6.3, H-2′b); 2.21 (ddd, 1H, J_{gem} = 13.4, $J_{2'a,1'}$ = 6.1, $J_{2'a,3'}$ = 3.6, H-2′a); 3.57 (dd, 1H, J_{gem} = 11.8, $J_{5'b,4'}$ = 4.0, H-5′b); 3.63 (dd, 1H, J_{gem} = 11.8, $J_{5'a,4'}$ = 3.7, H-5′a); 3.82 (ddd, 1H, $J_{4',5'}$ = 4.0, 3.7, $J_{4',3'}$ = 3.6, H-4′); 4.23 (dt, 1H, $J_{3',2'}$ = 6.3, 3.6, $J_{3',4'}$ = 3.6, H-3′); 4.48

(bs, 2H, CH_2N); 4.67 (q, 2H, J_{vic} = 7.0, $CH_3C\mathbf{H_2}O$); 6.10 (dd, 1H, $J_{1',2'}$ = 7.0, 6.1, H-1′); 6.96 (bs, 2H, NH_2); 8.10 (s, 1H, H-6); 8.74 (bs, 1H, NH). ^{13}C NMR (125.7 MHz, DMSO-d_6, T = 100 °C): 15.17 ($\mathbf{C}H_3CH_2O$); 33.84 (CH_2N); 40.66 (CH_2-2′); 61.00 (CH_2-5′); 68.75 ($CH_3\mathbf{C}H_2O$); 70.00 (CH-3′); 76.28 (cyt-**C**≡C-CH_2); 85.50 (CH-1′); 87.42 (CH-4′); 98.66 (C-5); 90.13 (cyt-C≡**C**-CH_2); 144.22 (CH-6); 153.07 (C-2); 164.02 (C-4); 172.04 (C-4-cyclobut); 177.25 (C-3-cyclobut); 182.42, 188.75 (C-1,2-cyclobut).
HR/MS (ESI$^+$) for $C_{18}H_{21}O_7 N_4$: $[M + H]^+$ calculated 405.14048, found 405.14049.

Synthesis of modified 2’-deoxycytidine triphosphate (dC^{ESQ}TP)

3-{[5- (3-Amino-1-propynyl) -2'-deoxycytidine]-5'-*O*-triphosphate}-4- ethoxycyclobut-3-ene-1,2-dione (dC^{ESQ}TP)

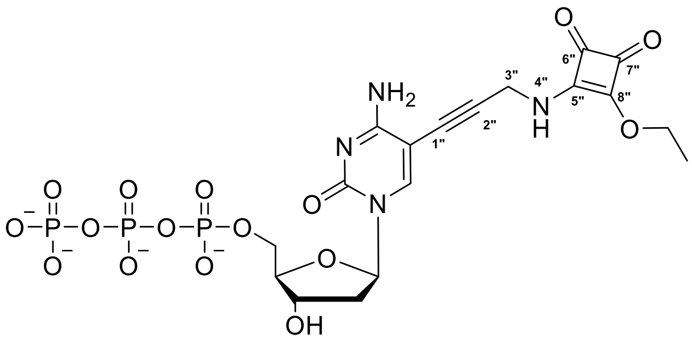

A mixture of two isomers A:B ~ 3:2

Ethoxy squarate modified nucleoside (**dC^{ESQ}**, 0.050 g, 0.124 mmol) was dried at 80°C for 2 hours in vacuo. After cooling on ice, $PO(OMe)_3$ (0.4 mL) and $POCl_3$ (14 μL) were added under argon atmosphere. The reaction mixture was stirred for 1 hour at 0°C. In a separate flask, the mixture of $(NHBu_3)H_2P_2O_7$ (0,370 g) and Bu_3N (90 μL) in dry DMF (1 mL) was prepared under argon atmosphere cooled to 0 °C and then added by the syringe to the reaction mixture. The mixture was stirred at 0°C 45 min. The reaction was stopped by addition of TEAB (2 M, 0.4 mL) and water (0.6 mL). The product was purified by C18 reversed-phase HPLC using water/methanol (5 to 50%) containing 0.1 M TEAB buffer as eluent. Several co-distillations with water and conversion to sodium salt (Dowex 50WX8 in Na^+ cycle) followed by freeze-drying from water gave the product **dC^{ESQ}TP** as white powder in 7% yield (0.006 g).
^{1}H NMR (500.0 MHz, D_2O, ref(dioxane) = 3.75 ppm): 1.40 – 1.46 (m, 6H, $C\mathbf{H_3}CH_2O$-A,B); 2.30 (dt, 2H, J_{gem} = 13.9, $J_{2'b,1'}$ = $J_{2'b,3'}$ = 6.4, H-2′b-A,B); 2.43 (ddd, 2H, J_{gem} = 13.9, $J_{2'a,1'}$ = 6.4, $J_{2'a,3'}$

= 4.5, H-2′a-A,B); 4.17 – 4.27 (m, 6H, H-4′,5′-A,B); 4.55 (bs, 2H, H-3″-A); 4.61 (dt, 1H, $J_{3',2'}$ = 6.4, 4.5, $J_{3',4'}$ = 4.5, H-3′); 4.65 (bs, 2H, H-3″-B); 4.69 – 4.80 (m, 4H, $CH_3C\mathbf{H_2}O$-A,B); 6.23 (t, 2H, $J_{1',2'}$ = 6.4, H-1′-A,B); 8.22 (s, 2H, H-6-A,B). ^{13}C NMR (125.7 MHz, D_2O, ref(dioxane) = 69.3 ppm): 17.78, 17.80 ($\mathbf{C}H_3CH_2O$-A,B); 37.09 (CH_2-3″-B); 37.36 (CH_2-3″-A); 42.17 (CH_2-2′-A,B); 67.66 (d, $J_{C,P}$ = 4.7, CH_2-5′-A,B); 72.62 (CH-3′-A,B); 73.55 ($CH_3\mathbf{C}H_2O$-A,B); 77.96 (cyt-**C**≡C-CH_2-B); 78.03 (cyt-**C**≡C-CH_2-A); 88.34 (d, $J_{C,P}$ = 9.0, CH-4′-A,B); 89.05 (CH-1′-A,B); 93.19 (cyt-C≡**C**-CH_2-A); 93.62 (cyt-C≡**C**-CH_2-B); 94.55 (C-5-A,B); 148.09 (CH-6-A,B); 158.81 (C-2-A,B); 167.79 (C-4-A,B); 175.75 (C-5″-B); 175.89 (C-5″-A); 180.11 (C-8″-A); 180.76 (C-8″-B); 186.47, 186.56, 191.41, 191.80 (C-6″,7″-A,B). ^{31}P{^{1}H} NMR (202.4 MHz, D_2O): -21.62 (br, 2P, P_β); -10.58 (d, 2P, J = 19.4, P_α); –6.70 (br, 2P, P_γ).
HR/MS (ESI$^-$) for $C_{18}H_{22}O_{16}N_4P_3$: [M - H]$^-$ calculated 643.02491, found 643.02440.

Synthesis of modified 2’-deoxycytidine monophosphate (dC^{ESQ}MP) and its conjugates with Ac-Lys-OH and lysine containing tripeptide (Ac-Ala-Lys-Ala-NH_2) (dC^{ESQLys}MP, dC^{ESQ3pept}MP)

3-{[5- (3-Amino-1-propynyl) -2'-deoxycytidine]-5'-*O*-monophosphate}-4- ethoxycyclobut-3-ene-1,2-dione (dC^{ESQ}MP)

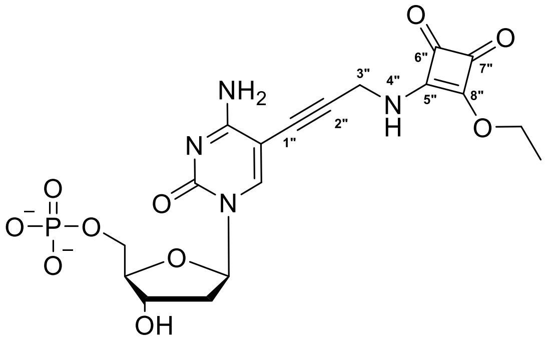

A mixture of two isomers A:B ~ 3:2

Ethoxy squarate modified nucleoside (**dC^{ESQ}**, 0.050 g, 0.124 mmol) was dried at 80°C for 2 hours in vacuo. After cooling on ice, $PO(OMe)_3$ (0.4 mL) and $POCl_3$ (14 µL) were added under argon atmosphere. The reaction mixture was stirred 2 hours at 0°C. The reaction was stopped by addition of TEAB (2 M, 0.4 mL) and water (0.6 mL). The product was purified by C18 reversed-phase HPLC using water/methanol (5 to 100%) containing 0.1 M TEAB buffer as eluent. Several co-distillations with water and conversion to sodium salt (Dowex 50WX8 in Na^+ cycle) followed by freeze-drying from water gave the product **dC^{ESQ}MP** as white powder in 37% yield (0.046 g).

^{1}H NMR (500.0 MHz, D_2O, ref(dioxane) = 3.75 ppm): 1.43 (t, 3H, J_{vic} = 7.0, C**H₃**CH₂O-B); 1.45 (t, 3H, J_{vic} = 7.0, C**H₃**CH₂O-A); 2.29 (dt, 2H, J_{gem} = 13.9, $J_{2'b,1'}$ = $J_{2'b,3'}$ = 6.5, H-2′b-A,B); 2.45 (ddd, 2H, J_{gem} = 13.9, $J_{2'a,1'}$ = 6.5, $J_{2'a,3'}$ = 3.9, H-2′a-A,B); 3.98 – 4.06 (m, 4H, H-5′-A,B); 4.18 (qd, 2H, $J_{4',3'}$ = $J_{4',5'}$ = 3.9, $J_{H,P}$ = 1.0, H-4′-A,B); 4.52 (dt, 1H, $J_{3',2'}$ = 6.5, 3.9, $J_{3',4'}$ = 3.9, H-3′); 4.55 (bs, 2H, H-3″-A); 4.65 (bs, 2H, H-3″-B); 4.69 – 4.79 (m, 4H, $CH_3C\mathbf{H_2}O$-A,B); 6.23 (t, 2H, $J_{1',2'}$ = 6.5, H-1′-A,B); 8.20 (s, 2H, H-6-A,B). ^{13}C NMR (125.7 MHz, D_2O, ref(dioxane) = 69.3 ppm): 17.72 ($\mathbf{C}H_3CH_2O$-B); 17.79 ($\mathbf{C}H_3CH_2O$-B); 37.06 (CH_2-3″-B); 37.31 (CH_2-3″-A); 42.30 (CH_2-2′-A,B); 66.77 (d, $J_{C,P}$ = 4.7, CH_2-5′-A,B); 73.38 (CH-3′-A,B); 73.56 ($CH_3\mathbf{C}H_2O$-A,B); 77.95 (cyt-**C**≡C-CH_2-B); 78.05 (cyt-**C**≡C-CH_2-A); 88.61 (d, $J_{C,P}$ = 8.6, CH-4′-A,B); 89.22 (CH-1′-A,B); 93.11 (cyt-C≡**C**-CH_2-A); 93.55 (cyt-C≡**C**-CH_2-B); 94.40 (C-5-A,B); 147.95 (CH-6-A,B); 158.67 (C-2-A,B); 167.66 (C-4-A,B); 175.66 (C-5″-B); 175.80 (C-5″-A); 180.14 (C-8″-A); 180.74 (C-8″-B); 186.41, 186.51, 191.34, 191.67 (C-6″,7″-A,B). ^{31}P{^{1}H} NMR (202.4 MHz, D_2O): 2.29 (B); 2.33 (A).

HR/MS (ESI$^-$) for $C_{18}H_{20}O_{10}N_4P$: [M - H]$^-$ calculated 483.09225, found 483.09195.

N^2-acetyl-N^6-(2-{[5- (3-Amino-1-propynyl) -2'-deoxycytidine]-5'-O-monophosphate}-3,4-dioxocyclobut-1-en-1-yl)lysine (dC^{ESQLys}MP)

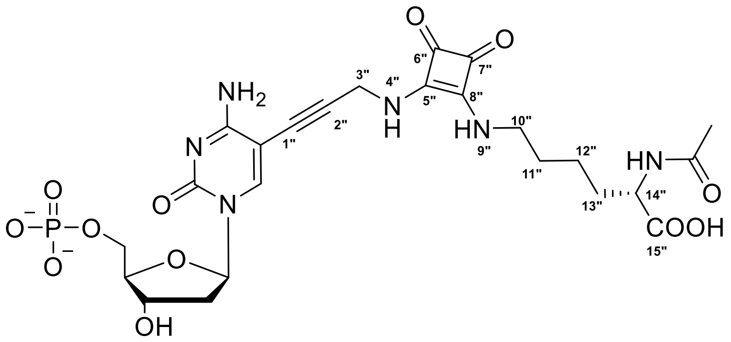

dC^{ESQ}MP (0.020 g, 0.041 mmol) and N_α-acetyl-L-lysine (0.015 g, 0.082 mmol) were dissolved in borate buffer (0.5 M, pH 9, 1.30 mL) and the mixture was stirred overnight at room temperature. The product was purified by C18 reversed-phase HPLC using water/methanol (5 to 100%) containing 0.1 M TEAB buffer as eluent. Several co-distillations with water and conversion to sodium salt (Dowex 50WX8 in Na^+ cycle) followed by freeze-drying from water gave the product **dC^{ESQLys}MP** in 54% yield (0.014 g).

^{1}H NMR (500.0 MHz, D_2O, ref(dioxane) = 3.75 ppm): 1.33 – 1.44 (m, 2H, H-12″); 1.58 – 1.71 (m, 3H, H-11″,13″b); 1.80 (m, 1H, H-13″a); 1.99 (s, 3H, CH_3CO); 2.32 (dt, 1H, J_{gem} = 13.9, $J_{2'b,1'}$

= $J_{2'b,3'}$ = 6.3, H-2′b); 2.44 (ddd, 1H, J_{gem} = 13.9, $J_{2'a,1'}$ = 6.3, $J_{2'a,3'}$ = 4.7, H-2′a); 3.55 – 3.64 (m, 2H, H-10″); 3.94 – 4.03 (m, 2H, H-5′); 4.10 (dd, 1H, $J_{14'',13''}$ = 9.1, 4.5, H-14″); 4.15 (qd, 1H, $J_{4',3'}$ = $J_{4',5'}$ = 3.9, $J_{H,P}$ = 1.2, H-4′); 4.55 (ddd, 1H, $J_{3',2'}$ = 6.3, 4.7, $J_{3',4'}$ = 3.9, H-3′); 4.59, 4.63 (2 × d, 2 × 1H, J_{gem} = 17.7, H-3″); 6.23 (t, 1H, $J_{1',2'}$ = 6,3, H-1′); 8.38 (s, 1H, H-6). ^{13}C NMR (125.7 MHz, D_2O, ref(dioxane) = 69.3 ppm): 24.55 (**C**H_3CO); 24.87 (CH_2-12″); 32.59 (CH_2-11″); 33.81 (CH_2-13″); 37.14 (CH_2-3″); 42.32 (CH_2-2′); 46.82 (CH_2-10″); 57.85 (CH-14″); 65.85 (br, CH_2-5′); 72.82 (CH-3′); 78.56 (cyt-**C**≡C-CH_2); 88.93 (d, $J_{C,P}$ = 8.6, CH-4′); 88.96 (CH-1′); 93.78 (cyt-C≡**C**-CH_2); 94.50 (C-5); 148.62 (CH-6); 158.73 (C-2); 167.40 (C-4); 170.22 (C-5″); 171.39 (C-8″); 176.23 (CH_3**C**O); 182.23 (C-15″); 183.78, 184.54 (C-6″,7″). ^{31}P{^{1}H} NMR (202.4 MHz, D_2O): 4.44. HR/MS (ESI$^-$) for $C_{24}H_{29}O_{12}N_6NaP$: [M – 2H + Na]$^-$ calculated 647.14842, found 647.14740.

[(2R,3S,5R)-5-(5-{3-({2-({5-(2-acetamidopropanamido)-6-({1-amino-1-oxopropan-2-yl}amino)-6-oxohexyl}amino)-3,4-dioxocyclobut-1-en-1-yl}amino)prop-1-yn-1-yl}-4-amino-2-oxopyrimidin-1(2H)-yl)-3-hydroxytetrahydrofuran-2-yl]methyl phosphate (dC^{ESQ3pept}MP)

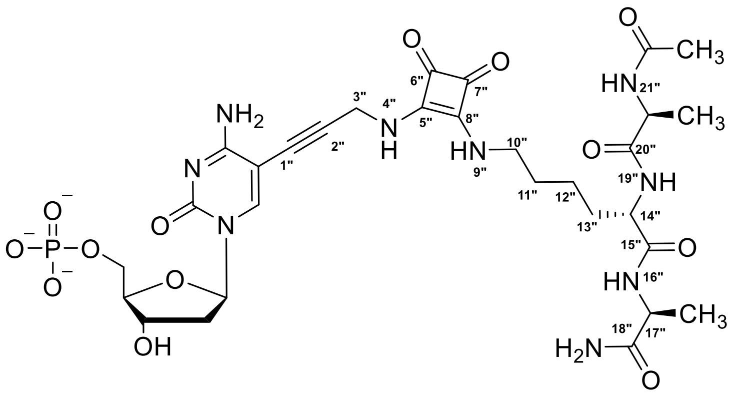

dC^{ESQ}MP (0.010 g, 0.021 mmol) and a tripeptide (0.013 g, 0.041mmol; for sequence see Table S3) were dissolved in borate buffer (0.5 M, pH 9; 0,650 mL) and the mixture was stirred overnight at room temperature. The product was purified by C18 reversed-phase HPLC using water/methanol (0 to 100%) containing 0.1 M TEAB buffer as eluent. Several co-distillations with water and conversion to sodium salt (Dowex 50WX8 in Na^+ cycle) followed by freeze-drying from water gave the product **dC^{ESQ3pept}MP** as white powder in 63% yield (0.010 g).

^{1}H NMR (500.0 MHz, D_2O, ref(dioxane) = 3.75 ppm): 1.33 (d, 3H, $J_{CH3,21''}$ = 7.2, CH_3-21″); 1.37 (d, 3H, $J_{CH3,17''}$ = 7.2, CH_3-17″); 1.38 – 1.46 (bm, 2H, H-12″); 1.61 – 1.70 (bm, 2H, H-11″); 1.72 – 1.88 (bm, 2H, H-13″); 1.99 (s, 3H, CH_3CO); 2.33 (dt, 1H, J_{gem} = 13.6, $J_{2'b,1'}$ = $J_{2'b,3'}$ = 6.3, H-2′b); 2.44 (ddd, 1H, J_{gem} = 13.6, $J_{2'a,1'}$ = 6.3, $J_{2'a,3'}$ = 5.2, H-2′a); 3.59 – 3.66 (bm, 2H, H-10″); 3.94

– 4.06 (bm, 2H, H-5′); 4.15 (q, 1H, $J_{4′,3′}$ = $J_{4′,5′}$ = 3.5, H-4′); 4.21 (q, 1H, $J_{21″,CH3}$ = 7.2, H-21″); 4.24 (q, 1H, $J_{17″,CH3}$ = 7.2, H-17″); 4.25 (dd, 1H, $J_{14″,13″}$ = 8.9, 5.6, H-14″); 4.54 – 4.60 (m, 2H, H-3′,3″b); 4.64 (d, 1H, J_{gem} = 17.5, H-3″a); 6.24 (t, 1H, $J_{1′,2′}$ = 6.3, H-1′); 8.47 (bs, 1H, H-6). ^{13}C NMR (125.7 MHz, D_2O, ref(dioxane) = 69.3 ppm): 19.22 (CH_3-21″); 19.33 (CH_3-17″); 24.29 (**C**H_3CO); 24.49 (CH_2-12″); 32.34 (CH_2-11″); 32.86 (CH_2-13″); 37.18 (CH_2-3″); 42.48 (CH_2-2′); 46.68 (CH_2-10″); 52.22 (CH-17″); 52.63 (CH-21″); 56.27 (CH-14″); 65.63 (d, $J_{C,P}$ = 4.5, CH_2-5′); 72.51 (CH-3′); 74.45 (cyt-**C**≡C-CH_2); 88.91 (CH-1′); 88.98 (d, $J_{C,P}$ = 9.1, CH-4′); 93.61 (cyt-C≡**C**-CH_2); 94.49 (C-5); 148.98 (CH-6); 158.74 (C-2); 167.29 (C-4); 170.16 (C-5″); 171.46 (C-8″); 176.53 (C-15″); 176.88 (CH_3**C**O); 178.33 (C-20″); 180.30 (C-18″); 183.72, 184.39 (C-6″,7″). ^{31}P{^{1}H} NMR (202.4 MHz, D_2O): 4.39.

HR/MS (ESI$^-$) for $C_{30}H_{40}O_{13}N_9NaP$: [M + Na]$^-$ calculated 788.23864, found 788.23737.

2.3 General remarks for the biochemical part

All gels were analysed by fluorescence imaging using Typhoon FLA 9500 (GE Healthcare). PCR gels were visualized using transilluminator equipped with GBox iChemi-XRQ Bio imaging system (Syngene, Life Technologies). Mass spectra of oligonucleotides were measured on UltrafleXtreme MALDI-TOF/TOF (Bruker) mass spectrometer with 1 kHz smartbeam II laser. UV-Vis spectra were measured at room temperature on NanoDrop1000 (ThermoScientific). Fluorescence was measured on a Fluoromax 4 spectrofluorimeter (HORIBA Scientific). Samples were concentrated on CentriVap Vacuum Concentrator system (Labconco). Synthetic oligonucleotides (primers, templates and biotinylated templates; for sequences see Table S1) were purchased from Generi Biotech (Czech Republic). Natural nucleoside triphosphates (dATP, dGTP, dTTP, dCTP) BSA and histone human recombinant proteins (H2A, H2B, H3.1, H4) were purchased from New England Biolabs. KOD XL DNA polymerase and corresponding polymerase reaction buffer from Merck Millipore, streptavidine magnetic particles from Roche, QIAquick® Nucleotide Removal Kit from Qiagen. Milli-Q water was used for all experiments. PAGE stop solution used after PEX reactions contains: 80% [v/v] formamide, 20 mM EDTA, 0.025% [w/v] bromophenol blue and 0.025% [w/v] xylene cyanol. VPS loading buffer used for protein SDS gels contains: 0.05 M TRIS (pH 6.8), 17% glycerol, 16 mM mercaptoethanol, 3.5% SDS, bromophenol blue. PageBlue^{TM} protein staining solution from Thermofisher Scientific.

2.4 Synthesis of ESQ modified DNA and its conjugation with peptides

Incorporation of dC^{ESQ}TP into DNA by PEX

19 mer, 1 modification

The reaction mixture (20 μL) contained primer^{B} (3 μM, 1 μL), template^{19_1C} (3 μM, 1.5 μL), KOD XL DNA polymerase (0.25 U/μL, 0.25 μL), natural dGTP (0.4 mM, 0.5 μL), either natural or modified dCTP (0.4 mM, 1 μL) in enzyme reaction buffer (10X, 2 μL). Primer was labelled on its 5´-end by 6-carboxyfluorescein (6-FAM). The reaction mixture was incubated for 30 min at 60°C in a thermal cycler. Finished PEX reaction was stopped by addition of PAGE stop solution and heated for 5 min at 95°C prior to loading. Samples were separated by 12.5% PAGE

(acrylamide/bisacrylamide 19:1, 25% urea) under denaturing conditions (TBE 1×, 42 mA, 1 hour). Visualization was performed by fluorescence imaging (Figure S1 a).

20 mer, 1 modification

The reaction mixture (20 µL) contained primer^{A} (3 µM, 1 µL), template^{20_1C} (3 µM, 1.5 µL), KOD XL DNA polymerase (0.25 U/µL, 0.25 µL), natural dNTPs (dGTP, dATP, dTTP; 0.4 mM, 0.5 µL), either natural or modified dCTP (0.4 mM, 1 µL) in enzyme reaction buffer (10X, 2 µL). Primer was labelled on its 5′-end by 6-carboxyfluorescein (6-FAM). The reaction mixture was incubated for 30 min at 60°C in a thermal cycler. Finished PEX reaction was stopped by addition of PAGE stop solution and heated for 5 min at 95°C prior to loading. Samples were separated by 12.5% PAGE (acrylamide/bisacrylamide 19:1, 25% urea) under denaturing conditions (TBE 1×, 42 mA, 1 hour). Visualization was performed by fluorescence imaging (Figure S1 b).

31 mer, 4 modifications

The reaction mixture (20 µL) contained primer^{B} (3 µM, 1 µL), template^{31_4C} (3 µM, 1.5 µL), KOD XL DNA polymerase (0.25 U/µL, 0.3 µL), natural dNTPs (dGTP, dATP, dTTP; 4 mM, 0.7 µL), either natural or modified dCTP (4 mM, 0.7 µL) in enzyme reaction buffer (10X, 2 µL). Primer was labelled on its 5′-end by 6-carboxyfluorescein (6-FAM). The reaction mixture was incubated for 30 min at 60°C in a thermal cycler. Finished PEX reaction was stopped by addition of PAGE stop solution and heated for 5 min at 95°C prior to loading. Samples were separated by 12.5% PAGE (acrylamide/bisacrylamide 19:1, 25% urea) under denaturing conditions (TBE 1×, 42 mA, 1 hour). Visualization was performed by fluorescence imaging (Figure S1 b).

98 mer, 18 modifications

The reaction mixture (150 µL) contained prim$^{REV_LT25\text{-}TH}$ (100 µM, 18 µL), temp$^{FVL\text{-}A}$ (100 µM, 15 µL), KOD XL DNA polymerase (2.5 U/µL, 7.5 µL), natural dNTPs (dGTP, dATP, dTTP; 4 mM, 18.8 µL), either natural or modified dCTP (4 mM, 18.8 µL) in enzyme reaction buffer (10X, 15 µL). Primer was labelled on its 5′-end by 6-carboxyfluorescein (6-FAM). The reaction mixture was incubated for 5 min at 95°C, 1.5 min at 50°C and then 2 h at 60°C in a thermal cycler. Finished PEX reaction was stopped by addition of PAGE stop solution and heated for 5 min at 95°C prior to loading. Samples were separated by 12.5% PAGE (acrylamide/bisacrylamide 19:1, 25% urea)

under denaturing conditions (TBE 1×, 42 mA, 1 hour). Visualization was performed by fluorescence imaging (Figure S1 c).

Preparation of ethoxy squarate-modified oligonucleotide (ON_C^{ESQ}) by PEX followed by magnetoseparation

19 mer, 1 modification

The reaction mixture (50 μL) contained KOD XL DNA polymerase (0.25 U/μL, 0.85 μL), primer^{B} (100 μM, 1.7 μL), 5′-biotinylated template^{19_1C} (100 μM, 1.7 μL), dNTPs (either natural or modified; 4 mM, 2.38 μL each) in KOD XL reaction buffer (10X, 5 μL). The reaction mixture was incubated in a thermal cycler for 30 min at 60°C and stopped by cooling to 4°C.

Streptavidine magnetic particles (Roche; 50 μL) were washed with binding buffer (3 × 200 μL; 10 mM Tris, 1 mM EDTA, 100 mM NaCl, pH 7.5). The PEX solution and binding buffer (200 μL) were added. The mixture was incubated for 30 min at 15°C and 1400 rpm. The magnetic beads were collected on a magnet (DynaMagTM-2, Invitrogen), and washed with wash buffer (3 × 300 μL; 10 mM Tris, 1 mM EDTA, 500 mM NaCl, pH 7.5) and water (4 × 300 μL). Then water (50 μL) was added and the sample was denatured for 2 min at 900 rpm and 55°C. The beads were collected on a magnet and the solution was transferred into a clean vial. The product was evaporated to dryness, then dissolved in the water and analysed by MALDI-TOF mass spectrometry (the results are in Table S4, for copy of mass spectra see Figure S11).

20 mer, 1 modification

The reaction mixture (100 μL) contained KOD XL DNA polymerase (2.5 U/ μL, 0.48 μL), primer^{A} (100 μM, 4 μL), 5′-biotinylated template^{20_1C} (100 μM, 4 μL), dNTPs (either natural or modified; 4mM, 0.375 μL each) in KOD XL reaction buffer (10X, 10 μL). The reaction mixture was incubated in a thermal cycler for 30 min at 60°C and stopped by cooling to 4°C.

Streptavidine magnetic particles (Roche; 100 μL) were washed with binding buffer (3 × 200 μL; 10 mM Tris, 1 mM EDTA, 100 mM NaCl, pH 7.5). The PEX solution and binding buffer (200 μL) were added. The mixture was incubated for 30 min at 15°C and 1400 rpm. The magnetic beads were collected on a magnet (DynaMagTM-2, Invitrogen), and washed with wash buffer (3 × 300 μL; 10 mM Tris, 1 mM EDTA, 500 mM NaCl, pH 7.5) and water (4 × 300 μL). Then water (50

μL) was added and the sample was denatured for 2 min at 900 rpm and 55°C. The beads were collected on a magnet and the solution was transferred into a clean vial. The product was evaporated to dryness, then dissolved in the water and analysed by MALDI-TOF mass spectrometry (the results are in Table S4, for copy of mass spectra see Figure S12).

PCR with squaramate-modified dCTP

Agarose gel electrophoresis

PCR product containing 6X DNA loading dye (60 mM EDTA, 10 mM Tris-HCl (pH 7.6), 60% glycerol, 0.03% bromphenol blue, 0.03% xylene cyanol FF; Thermo Scientific) was subjected to horizontal electrophoresis (Owl EasyCastB, Thermo Scientific) and analyzed on agarose gel (containing 0.5x TBE buffer, pH 8). The gel was run at 118 V for ca. 90–120 min. PCR product was visualized with GelRed (Biotium, 10 000X in H_2O) under UV in the GBox.

98-mer

The PCR mixture (20 μL) contained KOD XL DNA polymerase (2.5 U/μL, 1 μL), primer^{FOR_L20} (10 μM, 4 μL), primer$^{REV_LT25\text{-}TH}$ (10 μM, 4 μL), temp$^{FVL\text{-}A}$ (1 μM, 0.5 μL), natural dNTPs (4 mM, 0.15 μL of each), **dC^{ESQ}TP** (4 mM, 1 μL) and KOD XL reaction buffer (10X, 2 μL) supplied by the manufacturer.

PCR cycler was preheated to 80°C and 30 PCR cycles were run under following conditions: preheating for 3 min at 94°C, denaturation for 1 min at 95°C, annealing for 1 min at 53°C, extension for 1 min at 72°C followed by final extension step of 2 min at 75°C. The reaction was stopped by cooling to 4°C. The PCR product was analyzed on a 2% agarose gel in 0.5 × TBE running buffer (Figure S2).

235-mer

The PCR mixture (20 μL) contained KOD XL DNA polymerase (2.5 U/μL, 1 μL), primer^{FOR_235} (10 μM, 4 μL), primer^{REV_235} (10 μM, 4 μL), temp^{PCR_235} (1.9 μM, 0.145 μL), natural dNTPs (0.4 mM, 1.5 μL of each), **dC^{ESQ}TP** (0.4 mM, 1.5 μL) and KOD XL reaction buffer (10X, 2 μL) supplied by the manufacturer.

PCR cycler was preheated to 80°C and 40 PCR cycles were run under following conditions: preheating for 3 min at 94°C, denaturation for 1 min at 94°C, annealing for 1 min at 55°C, extension for 1 min at 72°C followed by final extension step of 5 min at 75°C. The reaction was stopped by cooling to 4°C. The PCR product was analyzed on a 1.3% agarose gel in 0.5 × TBE running buffer (Figure S2).

Reaction of DNA^{ESQ} with sulfo-Cy-5-amine (DNA_C^{ESQCy5})

Fluorescence meassurements

Natural DNA and **DNA_C^{ESQ}** were prepared by PEX in semi-preparative scale as described above. The products were purified on QIAquick Nucleotide Removal Kit (QIAGEN) eluted with water and evaporated to dryness and reconstituted in borate buffer (0.5 mM, pH 9). The reaction mixtures for sulfo-Cy-5-amine addition (10 μL) contained purified PEX product (0.2 nmol of natural or modified DNA) and sulfo-Cy-5-amine (13.5 mM, 1.48 μL). All samples were incubated on 37°C 36 h. The products were purified on QIAquick Nucleotide Removal Kit (QIAGEN) eluted with water and the fluorescence of the products was measured on spectrofluorimeter (water; λ_{ex}=646 nm, λ_{em}=662nm, Figure S3).

PAGE analysis

Natural DNA and **DNA_C^{ESQ}** were prepared by PEX in semi-preparative scale as described above. Primer was labelled on its 5´-end by 6-carboxyfluorescein (6-FAM). The products were purified on QIAquick Nucleotide Removal Kit (QIAGEN) and eluted with water. The reaction mixtures for sulfo-Cy-5-amine addition (10 μL) contained purified PEX product (0.2 nmol of natural or modified DNA) and sulfo-Cy-5-amine (13.5 mM, 1.48 μL). All samples were incubated on 37°C 36 h. The products were purified on QIAquick Nucleotide Removal Kit (QIAGEN) eluted with water evaporated to dryness and dilluted with mixture water/50% glycerol (2:1). Samples were heated for 5 min at 95°C and separated by 20% PAGE (acrylamide/bisacrylamide 19:1, 25% urea) under denaturing conditions (TBE 1×, 42 mA, 1 hour). Visualization was performed by fluorescence imaging (Figure S3).

Reaction of DNA_C^{ESQ} with lysine and lysine containing peptides (DNA_C^{ESQLys}, DNA_C^{ESQ3pept}, DNA_C^{ESQ10pept})

PAGE analysis

DNA_C^{ESQ} was prepared by PEX in semi-preparative scale as described above. Primer was labelled on its 5′-end by 6-carboxyfluorescein (6-FAM). The product was purified on QIAquick Nucleotide Removal Kit (QIAGEN) eluted with water and evaporated to dryness. The reaction mixture (20 μL) contained **DNA_C^{ESQ}** (4.3 μM) and Ac-Lys-OH, tri- or decapeptide (11 mM) in borate buffer (0.5 M, pH 9). The reaction was incubated 36 h at 37°C in a thermal cycler then evaporated to dryness and reconstituted in water (20 μL) and PAGE stop solution and heated for 5 min at 95°C. Samples were separated by 20% PAGE (acrylamide/bisacrylamide 19:1, 25% urea) under denaturing conditions (TBE 1×, 42 mA, 1 hour). Visualization was performed by fluorescence imaging (Figure S4).

MALDI-TOF analysis (*ON_C^{ESQLys}, ON_C^{ESQ3pept}, ON_C^{ESQ10pept}*)

DNA_C^{ESQ} was prepared by PEX in semi-preparative scale as described above. Primer was labelled on its 5′-end by biotin. The product was purified on QIAquick Nucleotide Removal Kit (QIAGEN) eluted with water and evaporated to dryness. The reaction mixture (20 μl) contained **DNA_C^{ESQ}** (0.2 nmol) and Ac-Lys-OH, tri- or decapeptide (22 mM) in borate buffer (0.5 M, pH 9). The reaction was incubated 36 h at 37°C in a thermal cycler followed by magnetoseparation and analysed by MALDI-TOF mass spektrometry (the results are in Table S4, for copies of mass spectrum see Figures S13-S15).

Cross-linking of DNA_C^{ESQ} and individual recombinant proteins (BSA, H2A, H2B, H3.1 and H4)

FAM labeled natural DNA and **DNA_C^{ESQ}** were prepared by PEX in semi-preparative scale as described above and purified on QIAquick Nucleotide Removal Kit (QIAGEN). 25 pmol of natural or modified DNA was incubated with 50 pmol of individual proteins (Table S5) in 10 μL of phosphate buffer (4.5 mM, ph 7.4) at 37°C for 36 h. The reaction was dilluted with 2X VPS loading buffer, denatured 2 min at 100°C prior to loading and analysed by 17.5% SDS denaturing PAGE

(acrylamide/methylenebisacrylamide 29:1; 1.92 M glycine, 0.25 M Tris, 0.1% SDS) at room temperature (230 V, 70 min). Visualization was performed by fluorescence imaging (Figure S6).

Alternatively, nonlabeled **DNA_C^{ESQ}** (483 pmol) was incubated with individual histone proteins (161 pmol) in 35 μL of phosphate buffer (4.5 mM, ph 7.4) at 37°C for 36 h. The reaction was dilluted with 2X VPS loading buffer, denatured 2 min at 100°C prior to loading and analysed by 17.5% SDS denaturing PAGE (acrylamide/methylenebisacrylamide 29:1; 1.92 M glycine, 0.25 M Tris, 0.1% SDS) at room temperature (230 V, 70 min) and stained with PageBlue™ protein staining solution (Thermofisher; Figure S7).

Cross-linking of DNA_C^{ESQ_19}, DNA_4C^{ESQ} and GSTp53CD

Modified DNA_C^{ESQ_19} and DNA_4C^{ESQ} were prepared by PEX as described above. The products were purified on QIAquick Nucleotide Removal Kit (QIAGEN) and eluted with water. The reaction mixtures for GSTp53CD protein binding (20 μL) were prepared from purified PEX (6 ng/μL, 10 μL), KCl (500 mM, 2 μL), DTT (2 mM, 2 μL), VP buffer (50 mM Tris, 0.1% Triton-X100, pH 7.6; 2 μL) and GSTp53CD stock solution (400 ng/μL in 25 mM HEPES pH 7.6, 200 mM KCl, 10% glycerol, 1 mM DTT, 1 mM benzamidine; 2 μL). Control samples were prepared using corresponding natural DNA. All samples were incubated for 36 h at 37°C. 2X VPS loading buffer was added and the mixture was denatured 2 min at 100°C prior to loading and analysed by 10% SDS-PAGE (0.025 M Tris, 0.192 M glycine, 0.1% SDS) at room temperature (100 V/40 min then 150 V/60 min). Visualization was performed by fluorescence imaging (Figure S9).

Cross-linking of 98-mer PEX product and H3.1 recombinant protein

FAM labeled natural and modified 98-mer PEX products were prepared as described above purified on QIAquick Nucleotide Removal Kit (QIAGEN) and eluted with water. 25 pmol of natural or modified DNA was incubated with 2 or 10 equiv of H3.1 protein in 10 μL of phosphate buffer (4.5 mM, ph 7.4) at 37°C 36 h. The reaction mixtures were dilluted with 2X VPS loading buffer, denatured 2 min at 100°C prior to loading and analysed by 12% SDS-PAGE

(acrylamide/methylenebisacrylamide 29:1; 1.92 M glycine, 0.25 M Tris, 0.1% SDS) at room temperature (42 mA, 80 min). Visualization was performed by fluorescence imaging (Figure S10).

Characterization of crosslinks between DNA_C^{ESQ} and individual recombinant proteins (H2A, H2B, H3.1 and H4) by mass spectrometry

DNA_C^{ESQ} containing one modification (Table S1) was cross-linked to histones (H2A, H2B, H3.1, H4) as described above. 10 uL of reaction mixture was injected onto bioZen Intact C4 column (Phenomenex) and separated by gradient of acetonitrile in water (both mobile phases modified by 0.1% Formic acid). The separation was carried out by LC system (I-class, Waters) coupled to Mass Spectrometer (Synapt G2, Waters) to acquire *m/z* by positive electrospray ionization. Raw mass spectra of chromatographic peak containing conjugate was combined, subtracted and deconvoluted by MaxEnt1[3] algorithm (for spectrum see Figure S16-S18).

3. Copies of MALDI-TOF mass spectra

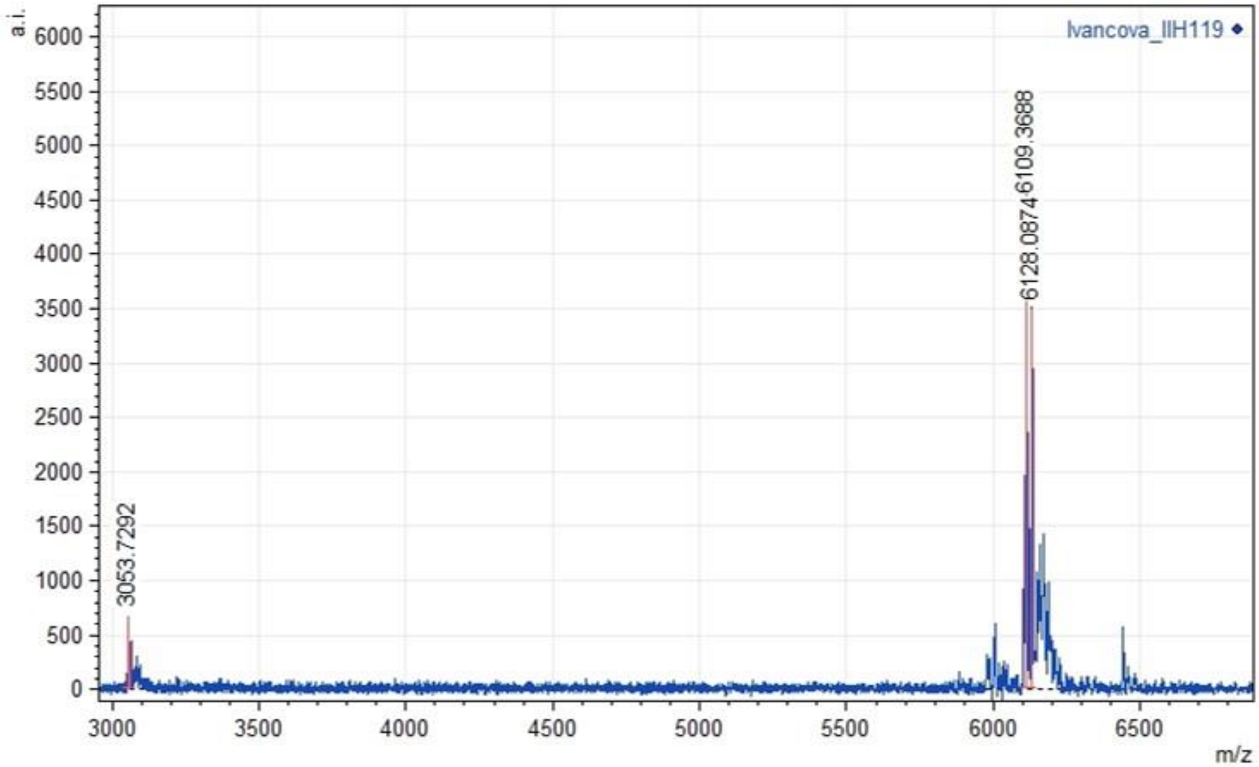

Figure S11. MALDI-TOF MS spectrum of **ON_C$^{\mathbf{ESQ_19}}$**, calculated for $[M+H]^+$: 6127.9 Da; found 6128.1 Da; the peak at *m/z* = 6109.4 can be assigned to the biotinylated template.

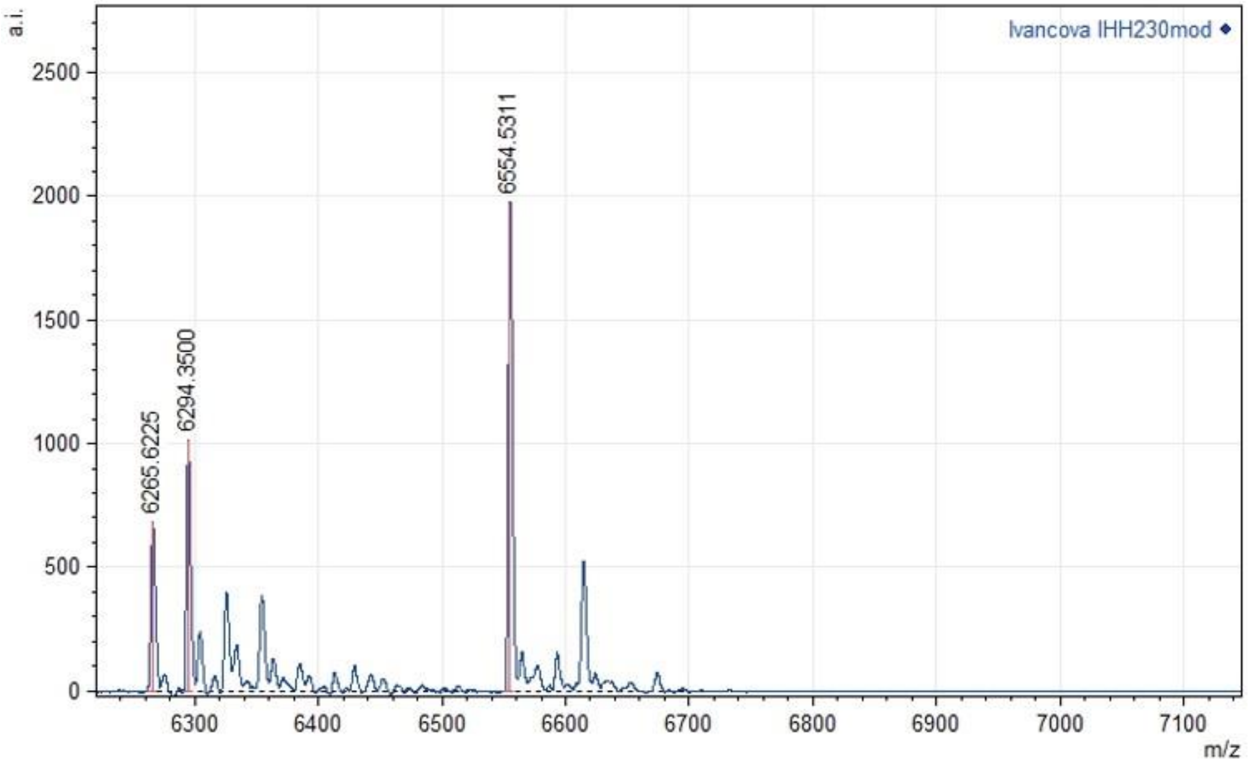

Figure S12. MALDI-TOF MS spectrum of **ON_C^{ESQ}**, calculated for $[M+H]^+$: 6293.3 Da; found 6294.3 Da; the peak at *m/z* = 6554.5 can be assigned to the biotinylated template and the peak at *m/z* = 6265.6 can be assigned to the hydrolyzed **ON_C^{ESQ}** [- CH_2CH_3 - H^+].

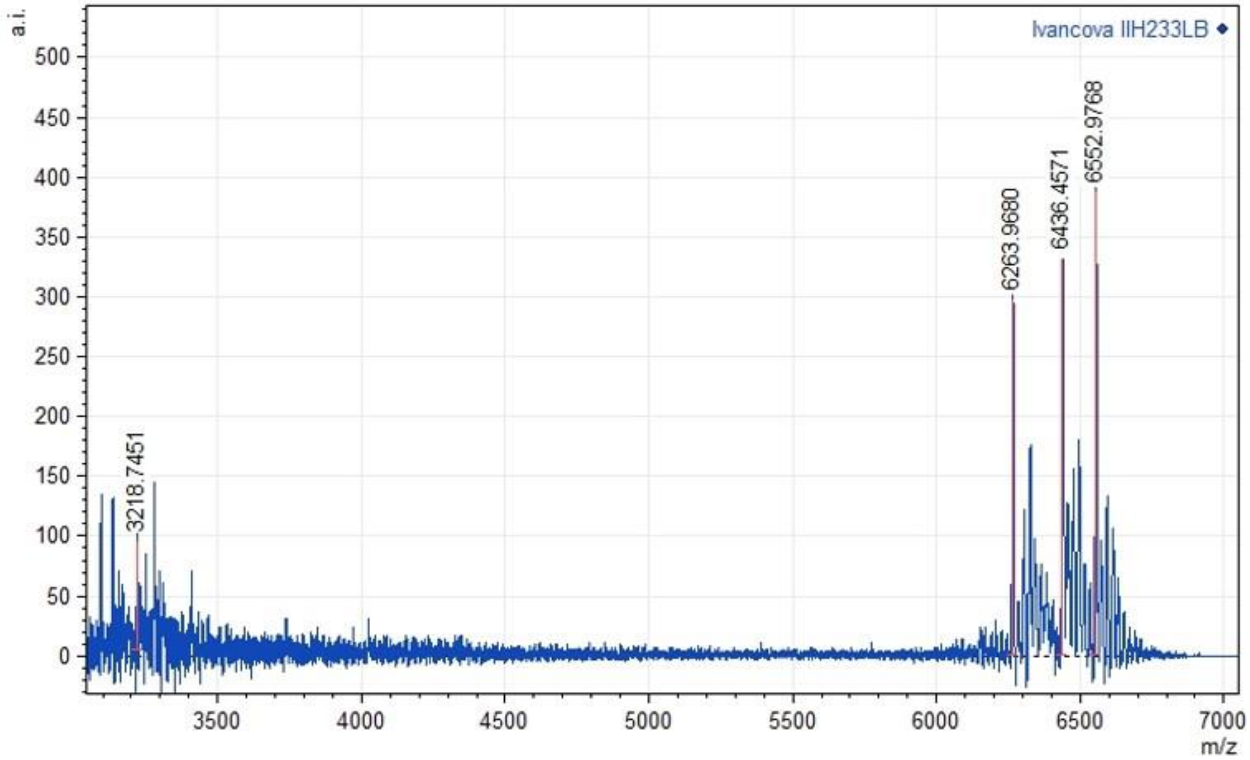

Figure S13. MALDI-TOF MS spectrum of **ON_C$^{\mathbf{ESQLys}}$**, calculated for $[M+H]^+$: 6435.5 Da; found 6436.4 Da; the peak at *m/z* = 6552.9 can be assigned to the biotinylated template and the peak at *m/z* = 6263.9 can be assigned to the hydrolyzed **ON_C$^{\mathbf{ESQ}}$** [- CH_2CH_3 - H^+].

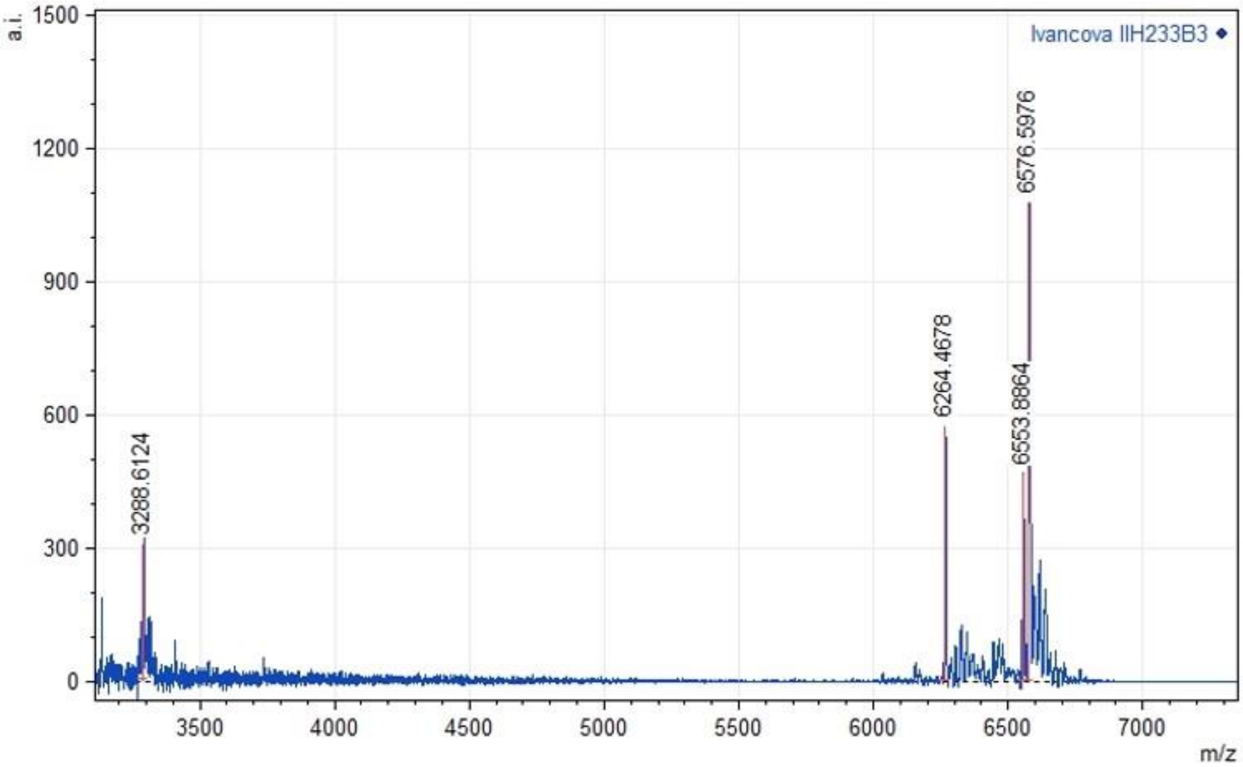

Figure S14. MALDI-TOF MS spectrum of **ON_C$^{\mathbf{ESQ3pept}}$**, calculated for $[M+H]^+$: 6576.5 Da; found 6576.6 Da; the peak at *m/z* = 6553.9 can be assigned to the biotinylated template and the peak at *m/z* = 6264.5 can be assigned to the hydrolyzed **ON_C$^{\mathbf{ESQ}}$** $[- CH_2CH_3 - H^+]$.

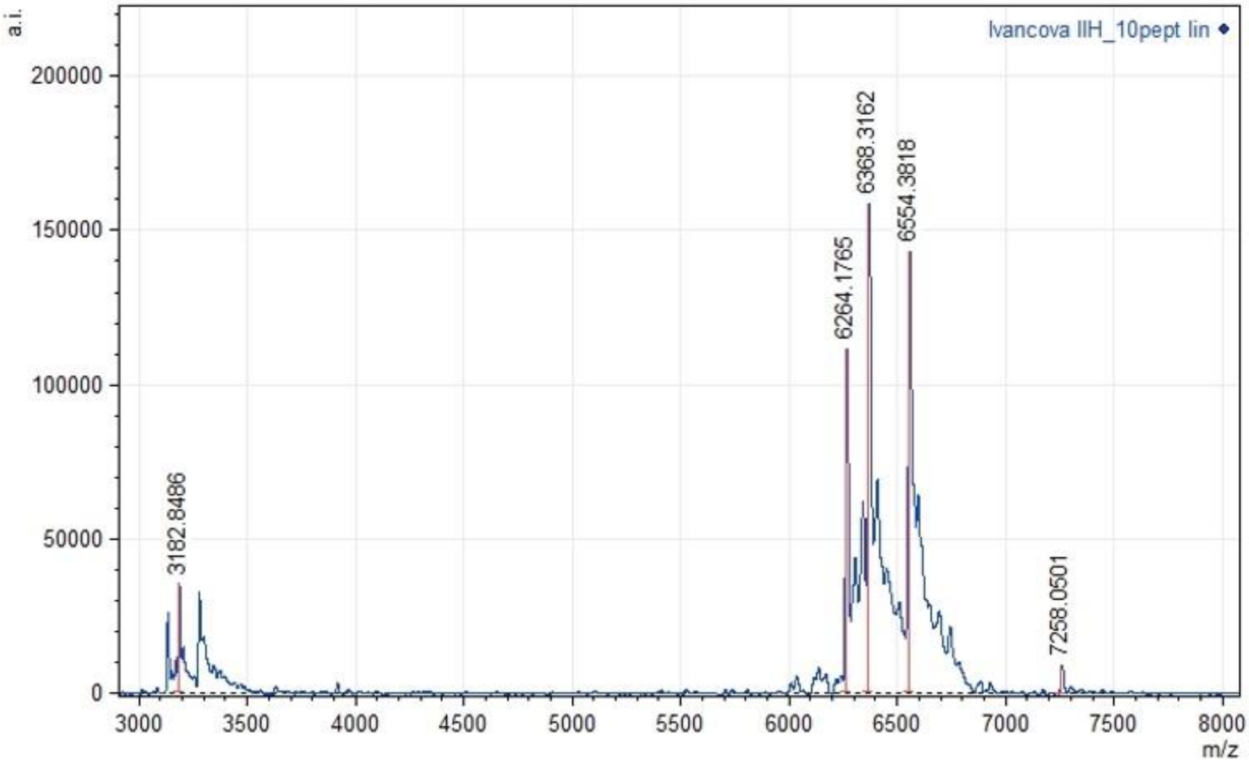

Figure S15. MALDI-TOF MS spectrum of **ON_C$^{\mathbf{ESQ10pept}}$**, calculated for $[M+H]^+$: 7257.8 Da; found 7258.0 Da; the peak at *m/z* = 6554.4 can be assigned to the biotinylated template, the peak at *m/z* = 6264.2 can be assigned to the hydrolyzed **ON_C$^{\mathbf{ESQ}}$** [- CH_2CH_3 - H^+] and the peak at 6368.3 Da can be assigned to the product of decomposition of peptide part of the conjugate **ON_C$^{\mathbf{ESQ10pept}}$** in MS.

4. Copies of MS spectra of Dna-protein cross-links

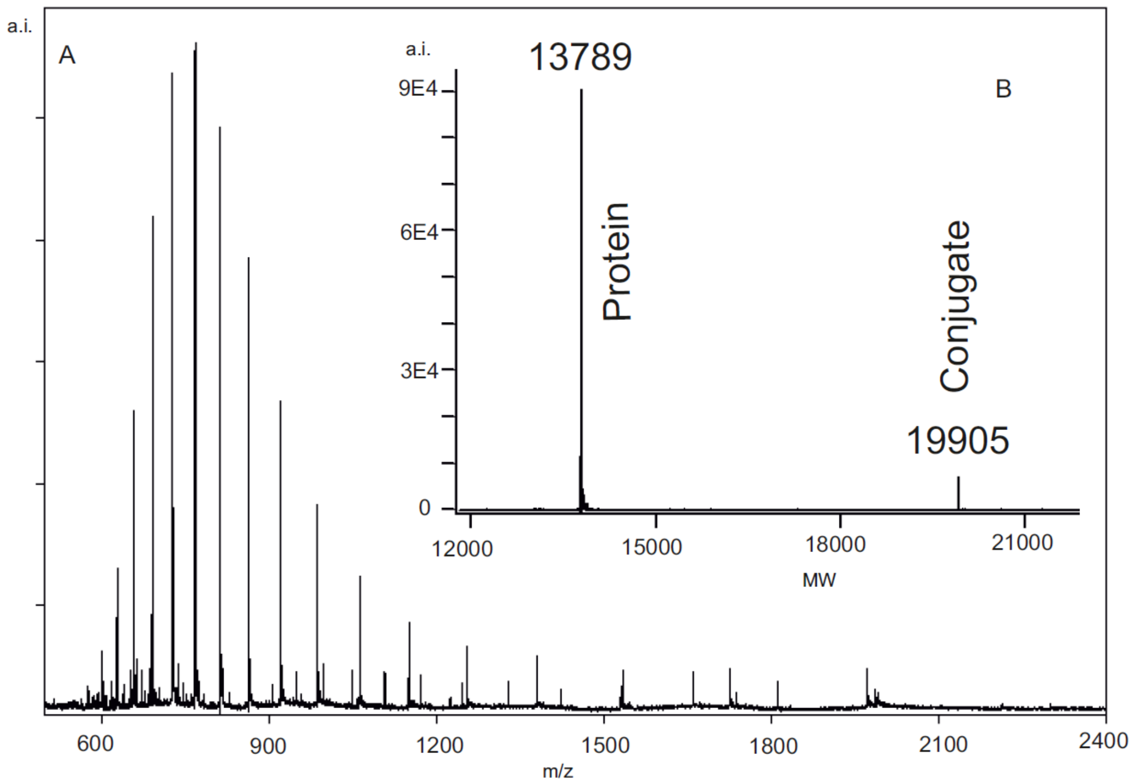

Figure S16. MS spectrum of chromatographic peak of histone H2B (M= 13789) cross-linked to **DNA_C^{ESQ}** (M= 6293.3 for single strand DNA with one modification; see Table S4). A) combined raw spectrum B) deconvoluted spectrum by MaxEnt1. The signal at 19905 corresponds to **ON_C^{ESQH2B}** conjugate (see Table S6).

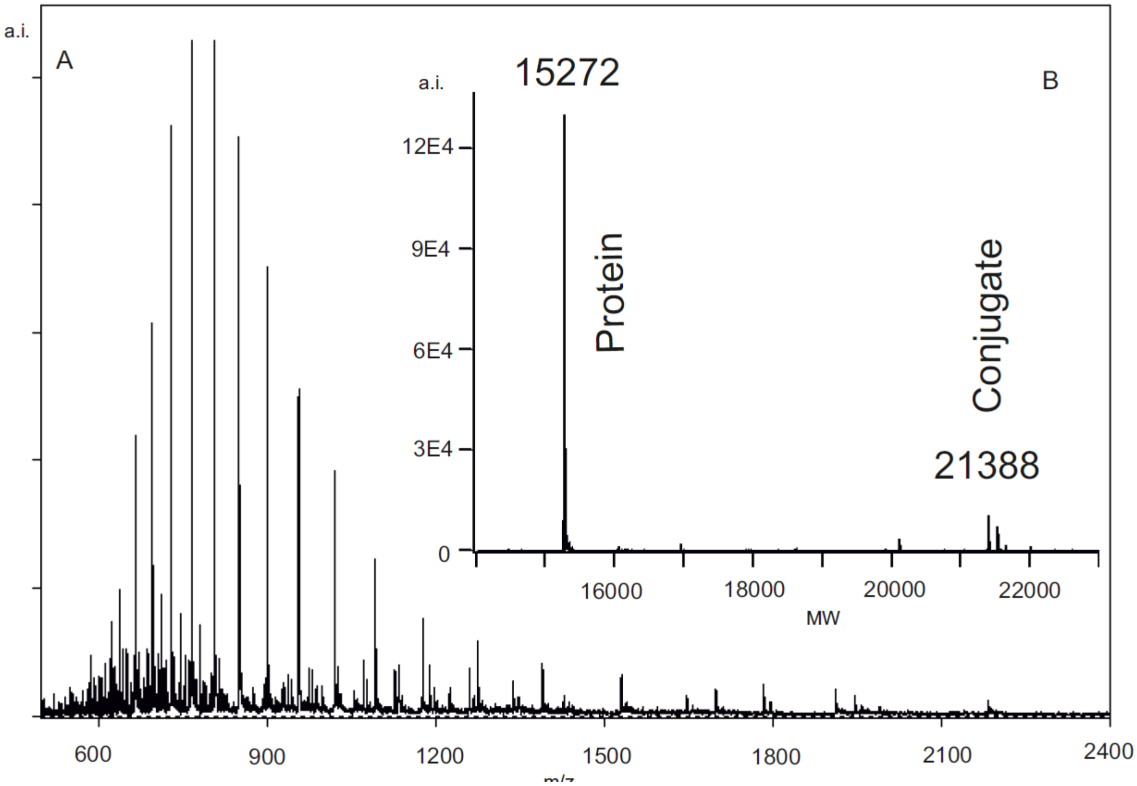

Figure S17. MS spectrum of chromatographic peak of histone H3.1 (M= 15273) cross-linked to **DNA_C^ESQ^** (M= 6293.3 for single strand DNA with one modification; see Table S4). A) combined raw spectrum B) deconvoluted spectrum by MaxEnt1. The signal at 21388 corresponds to **ON_C^ESQH3.1^** conjugate (see Table S6).

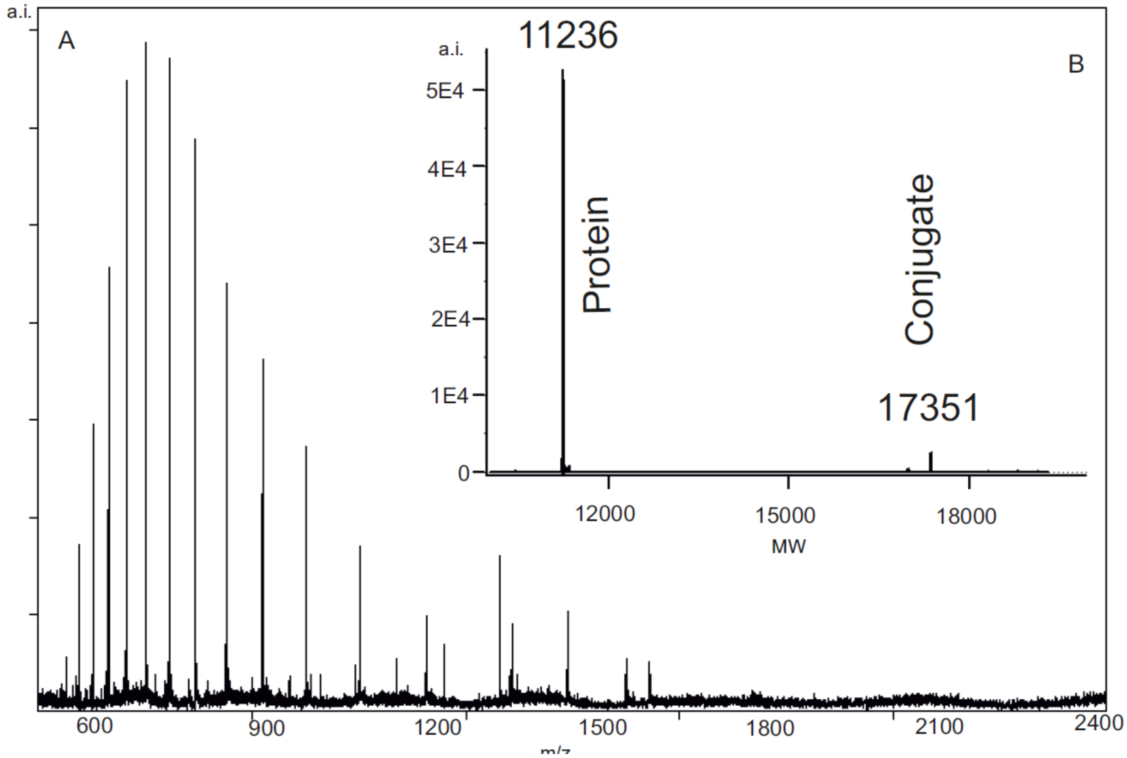

Figure S18. MS spectrum of chromatographic peak of histone H4 (M= 11236) cross-linked to **DNA_C$^{\mathbf{ESQ}}$** (M= 6293.3 for single strand with one modification; see Table S4). A) combined raw spectrum B) deconvoluted spectrum by MaxEnt1. The signal at 17351 corresponds to **ON_C$^{\mathbf{ESQH4}}$** conjugate (see Table S6).

5. Copies of NMR spectra

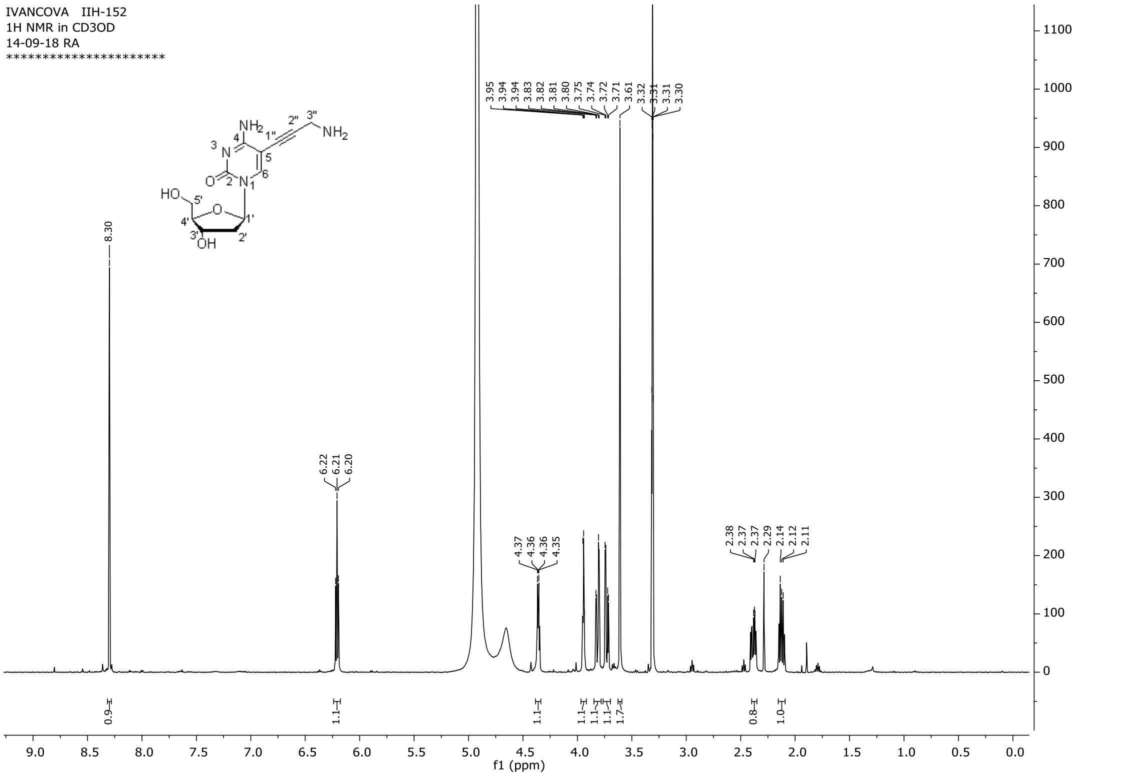

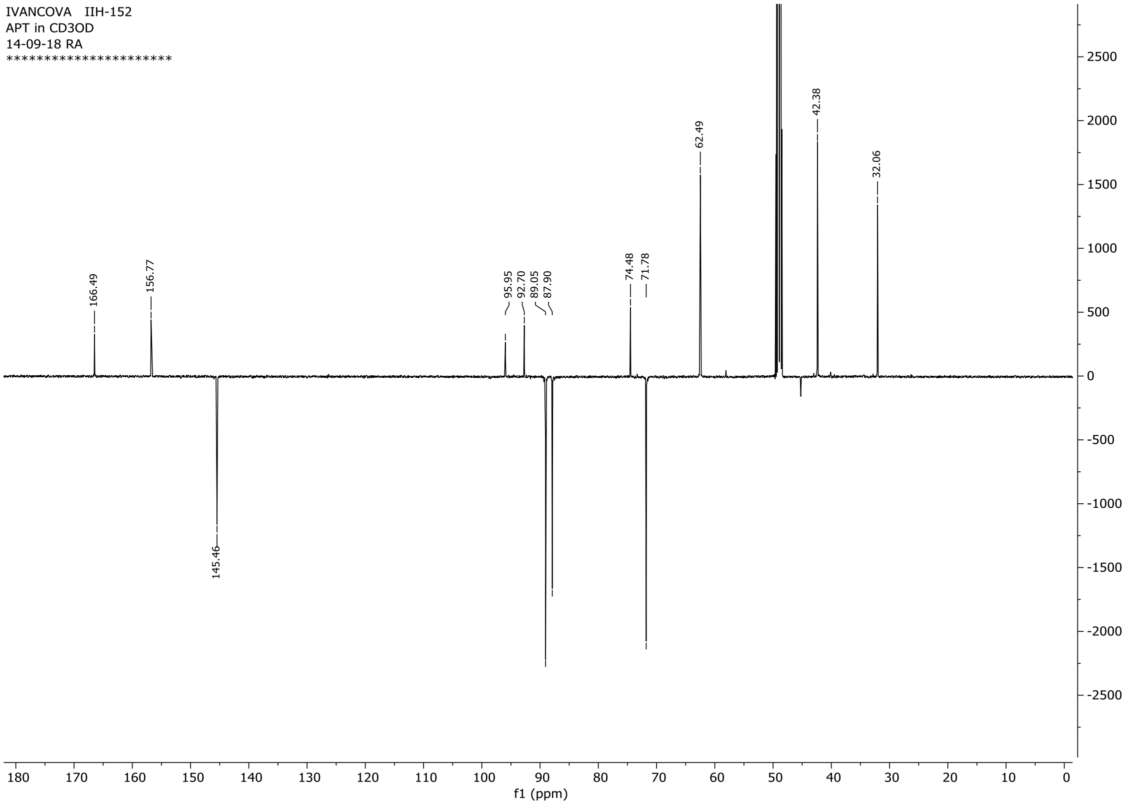

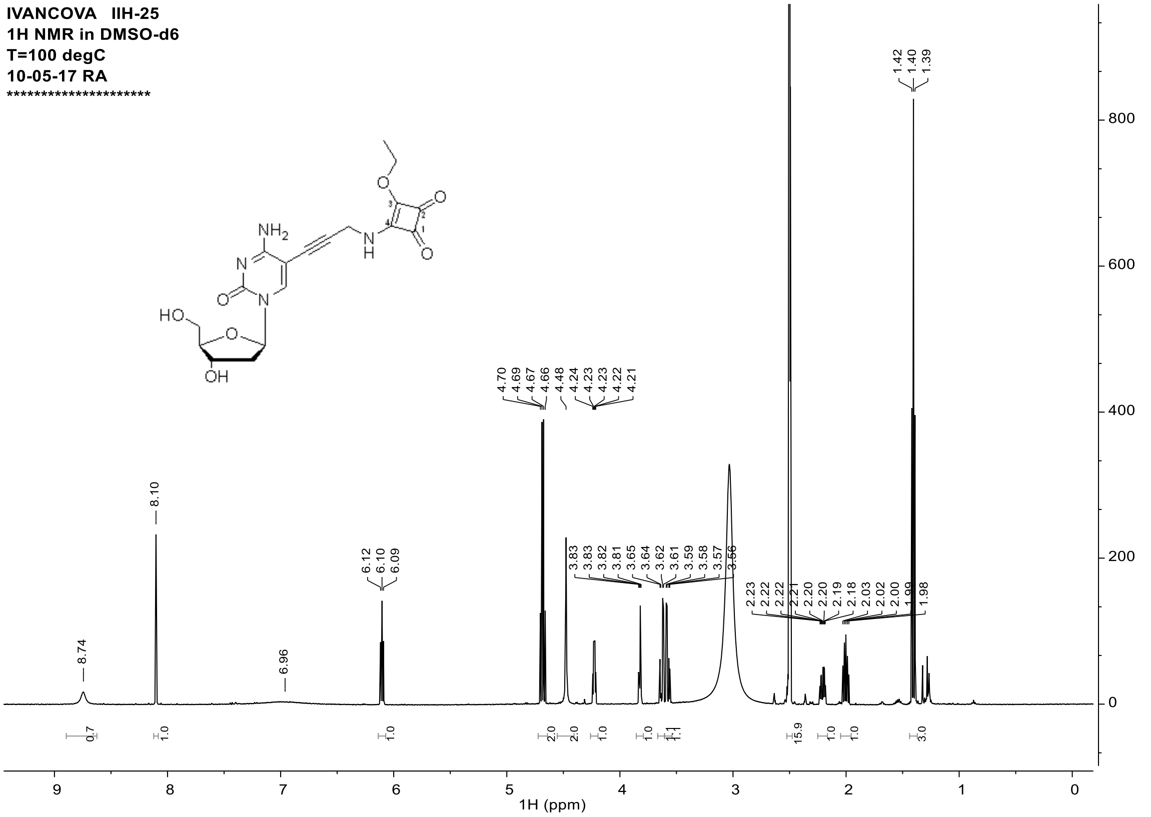
IVANCOVA IIH-25
1H NMR in DMSO-d6
T=100 degC
10-05-17 RA
****
1H (ppm)

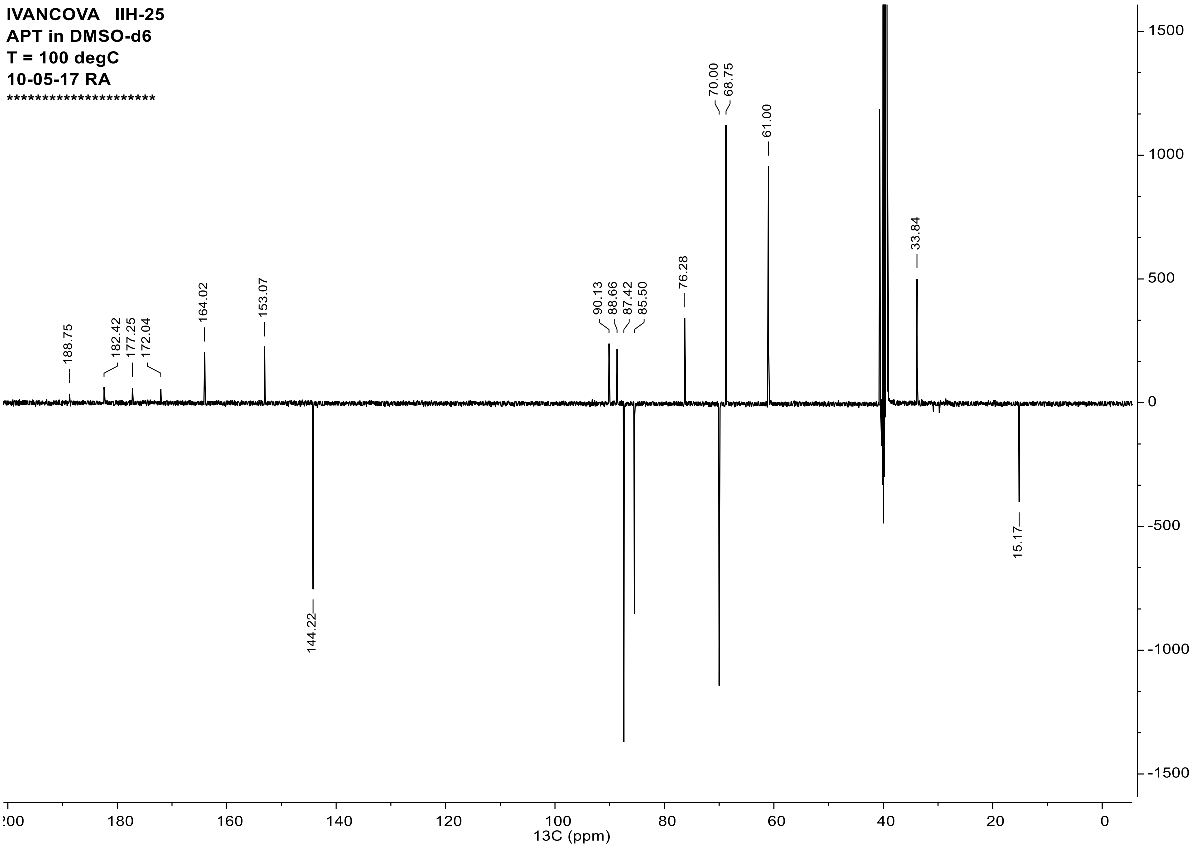
IVANCOVA IIH-25
APT in DMSO-d6
T = 100 degC
10-05-17 RA
****
13C (ppm)

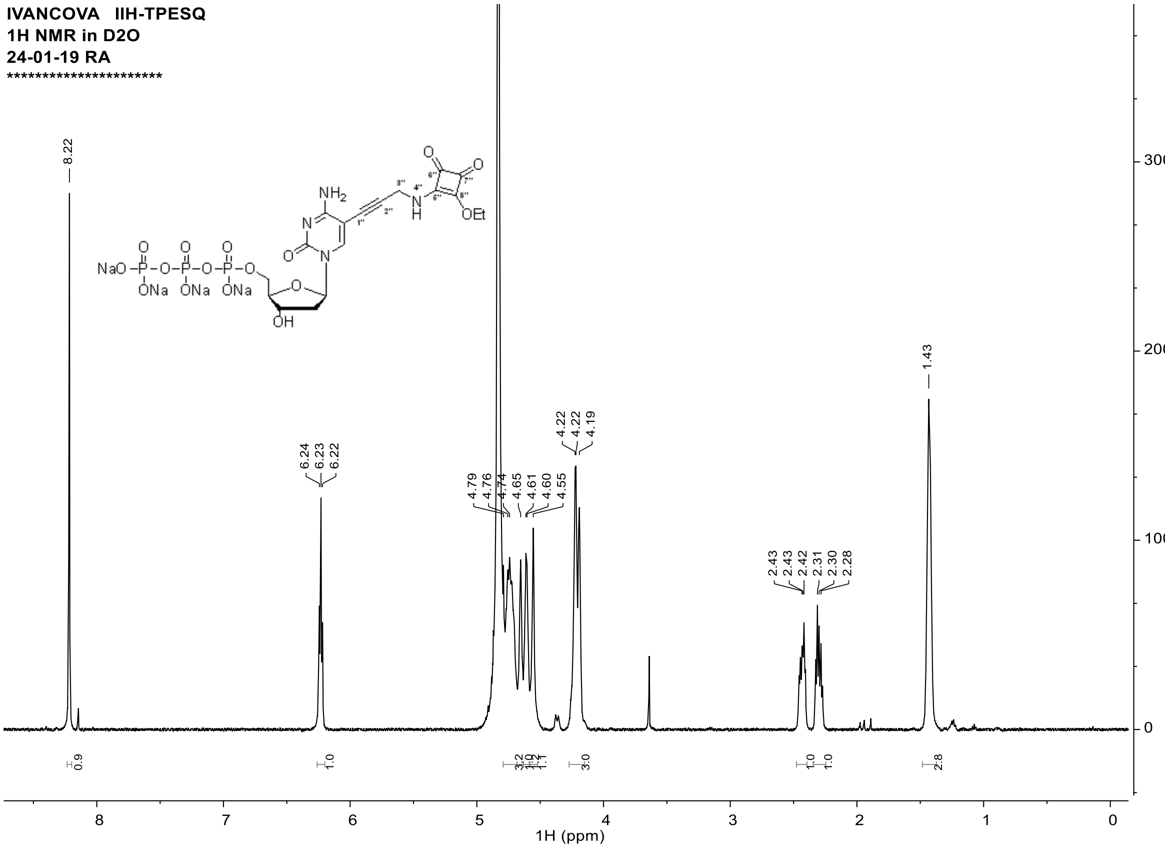
IVANCOVA IIH-TPESQ
1H NMR in D2O
24-01-19 RA
****
8.22
6.24
6.23
6.22
4.79
4.76
4.74
4.65
4.61
4.60
4.55
4.22
4.22
4.19
2.43
2.43
2.42
2.31
2.30
2.28
1.43
0.9
1.0
3.2
1.0
1.1
3.0
1.0
1.0
2.8
1H (ppm)

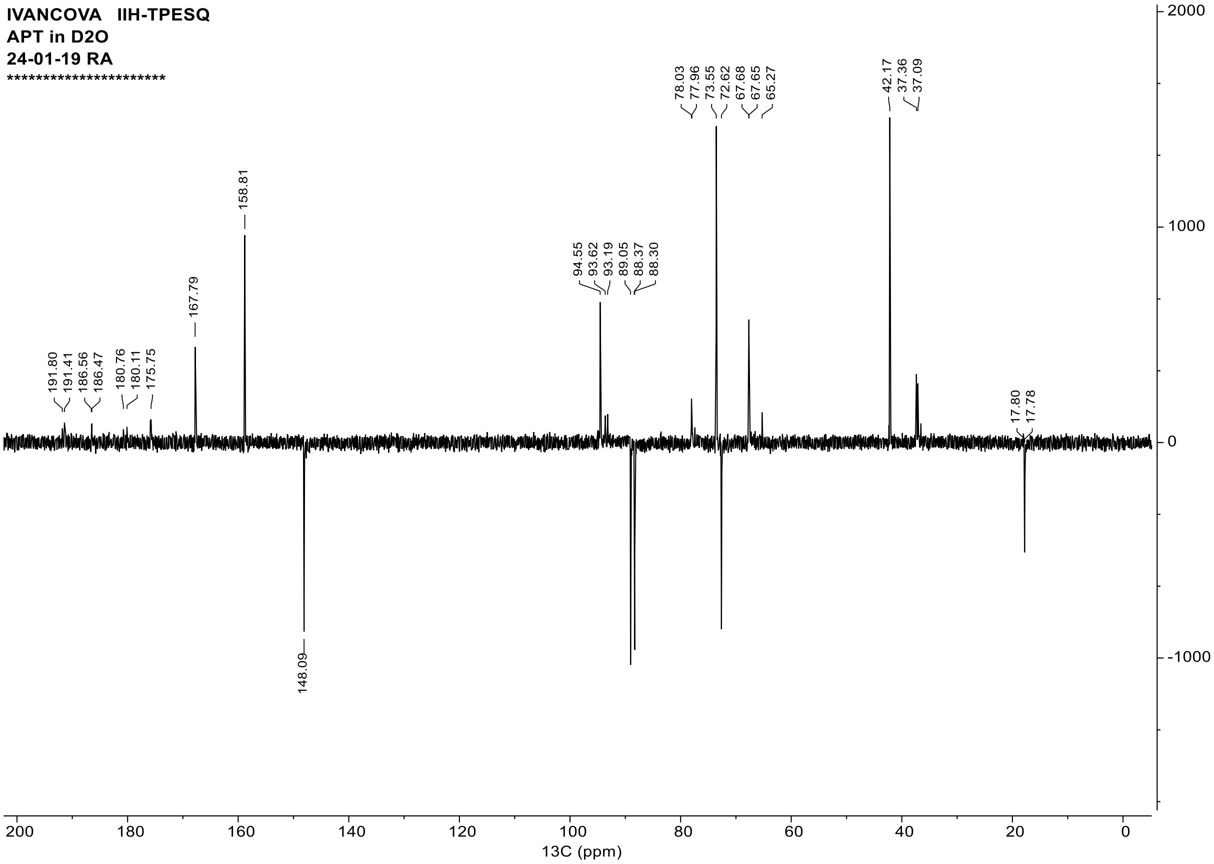
IVANCOVA IIH-TPESQ
APT in D2O
24-01-19 RA
****
191.80
191.41
186.56
186.47
180.76
180.11
175.75
167.79
158.81
148.09
94.55
93.62
93.19
89.05
88.37
88.30
78.03
77.96
73.55
72.62
67.68
67.65
65.27
42.17
37.36
37.09
17.80
17.78
13C (ppm)

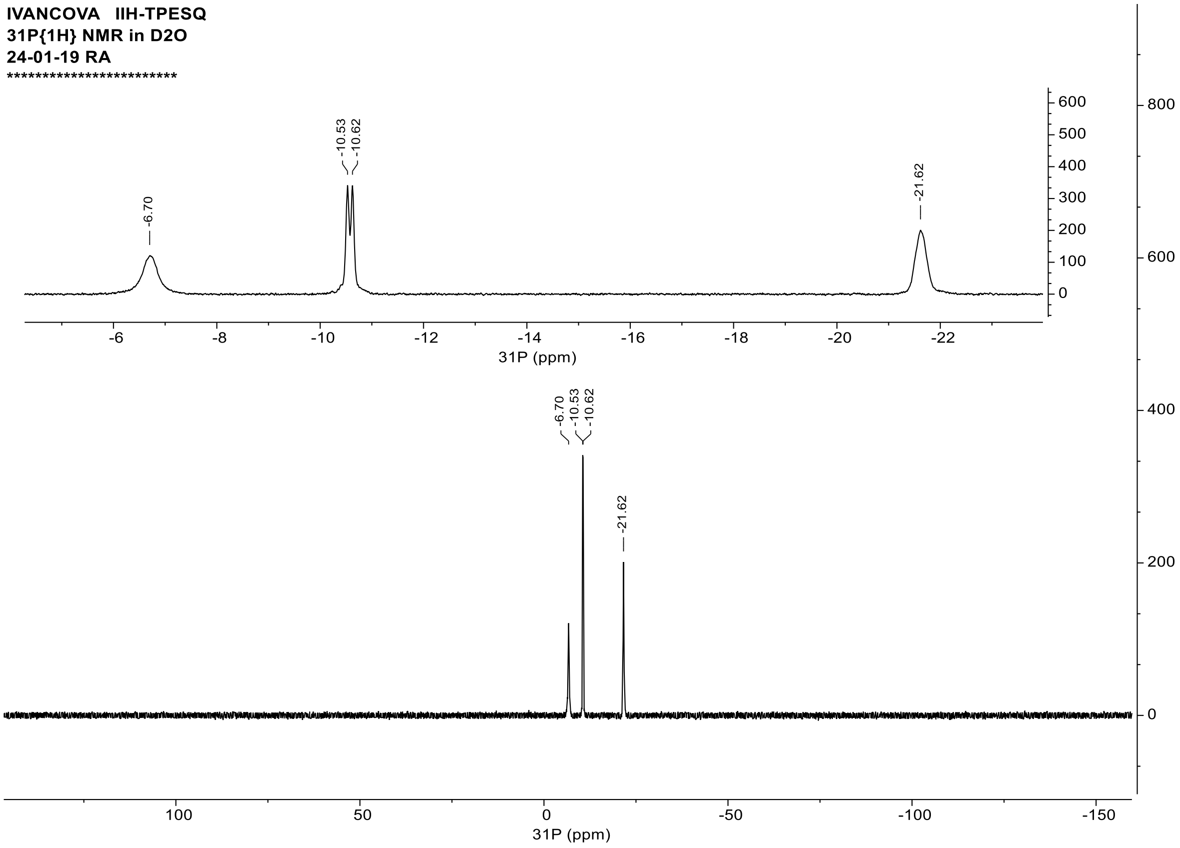

IVANCOVA IIH-TPESQ
31P{1H} NMR in D2O
24-01-19 RA
****
-6.70
-10.53
-10.62
-21.62
600
500
400
300
200
100
0
800
600
400
200
0
-6
-8
-10
-12
-14
-16
-18
-20
-22
31P (ppm)
100
50
0
-50
-100
-150
31P (ppm)

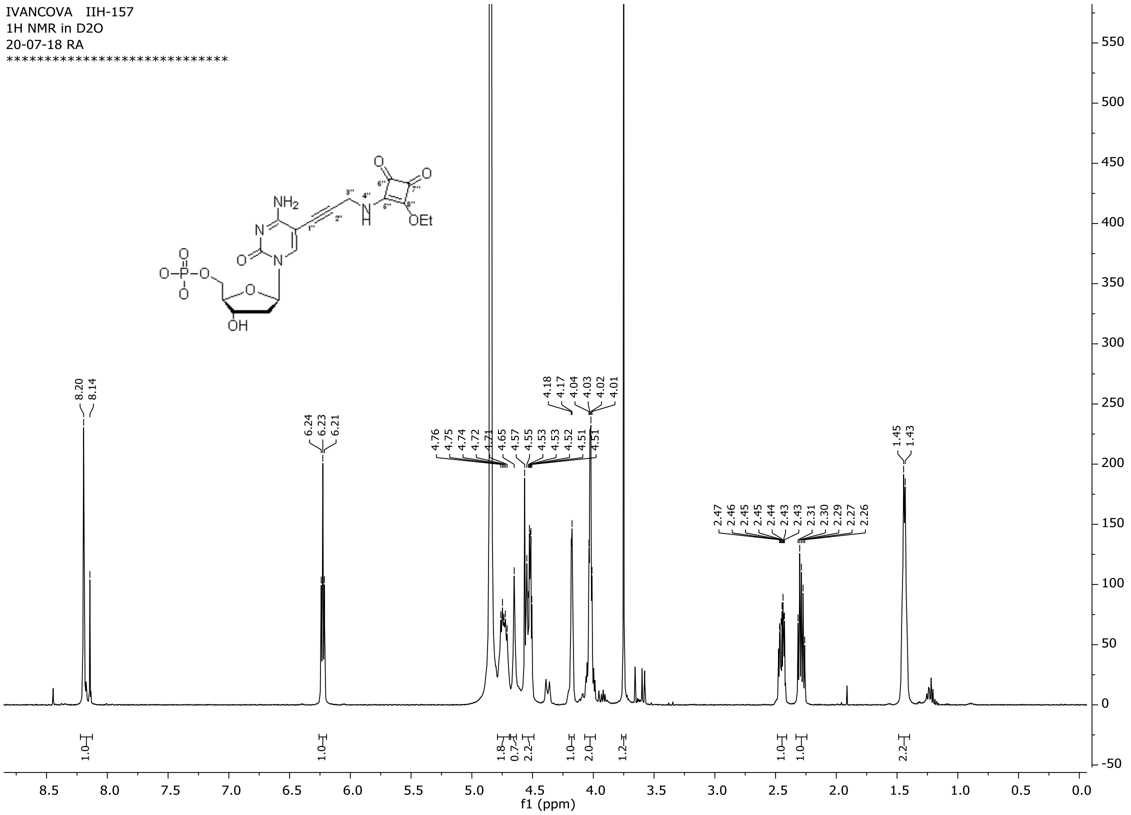
IVANCOVA IIH-157
1H NMR in D2O
20-07-18 RA
NH2
N
O
N
O
O-P-O
O
OH
OEt
H
N
8.20
8.14
6.24
6.23
6.21
4.76
4.75
4.74
4.72
4.71
4.65
4.57
4.55
4.53
4.53
4.52
4.51
4.51
4.18
4.17
4.04
4.03
4.02
4.01
2.47
2.46
2.45
2.45
2.44
2.43
2.43
2.31
2.30
2.29
2.27
2.26
1.45
1.43
1.0
1.0
1.8
0.7
2.2
1.0
2.0
1.2
1.0
1.0
2.2
f1 (ppm)

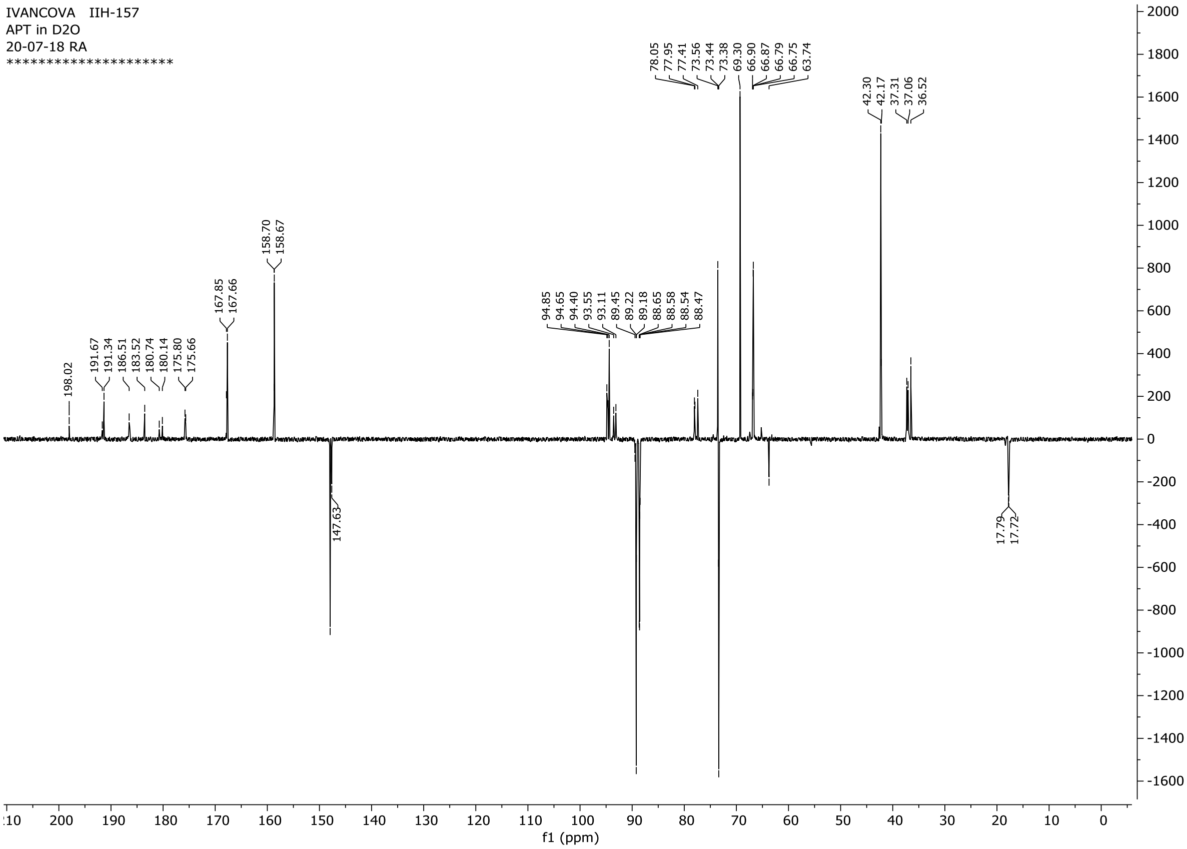
IVANCOVA IIH-157
APT in D2O
20-07-18 RA
198.02
191.67
191.34
186.51
183.52
180.74
180.14
175.80
175.66
167.85
167.66
158.70
158.67
147.63
94.85
94.65
94.40
93.55
93.11
89.45
89.22
89.18
88.65
88.58
88.54
88.47
78.05
77.95
77.41
73.56
73.44
73.38
69.30
66.90
66.87
66.79
66.75
63.74
42.30
42.17
37.31
37.06
36.52
17.79
17.72
f1 (ppm)

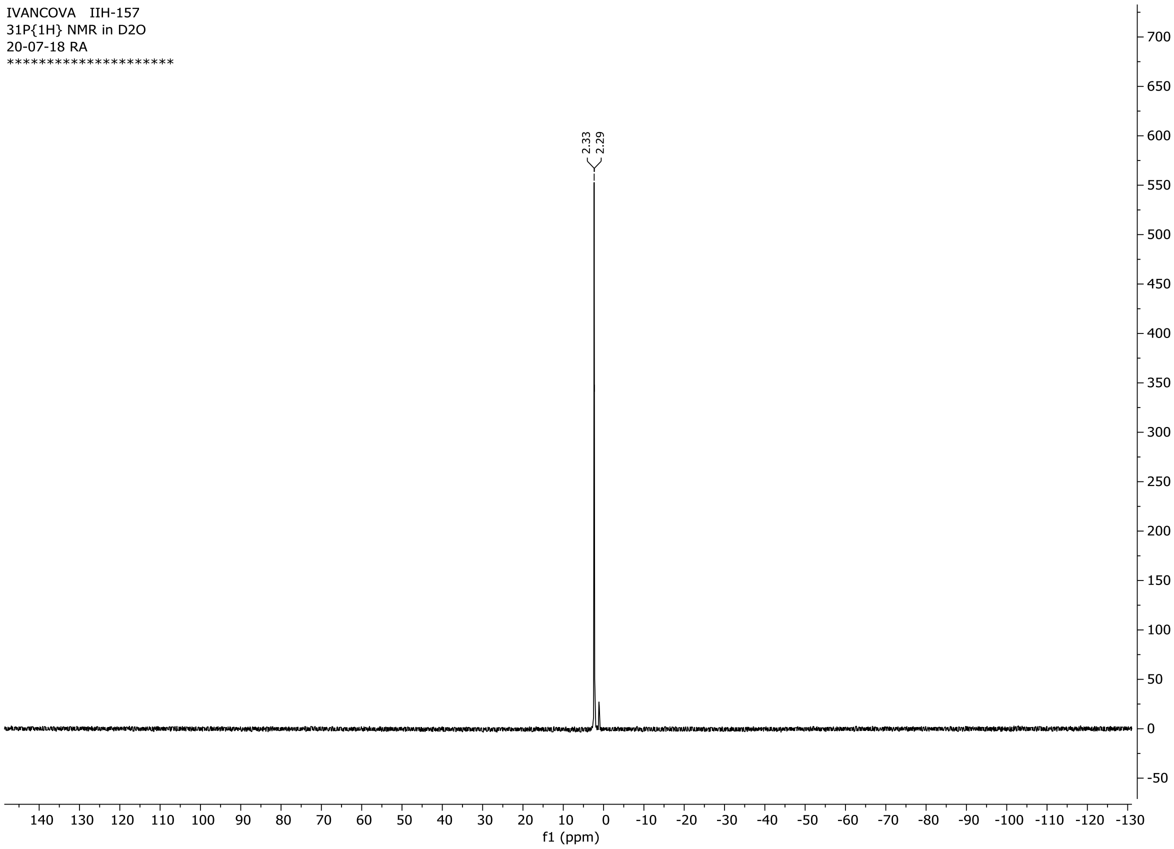
IVANCOVA IIH-157
31P{1H} NMR in D2O
20-07-18 RA
****
2.33
2.29
f1 (ppm)

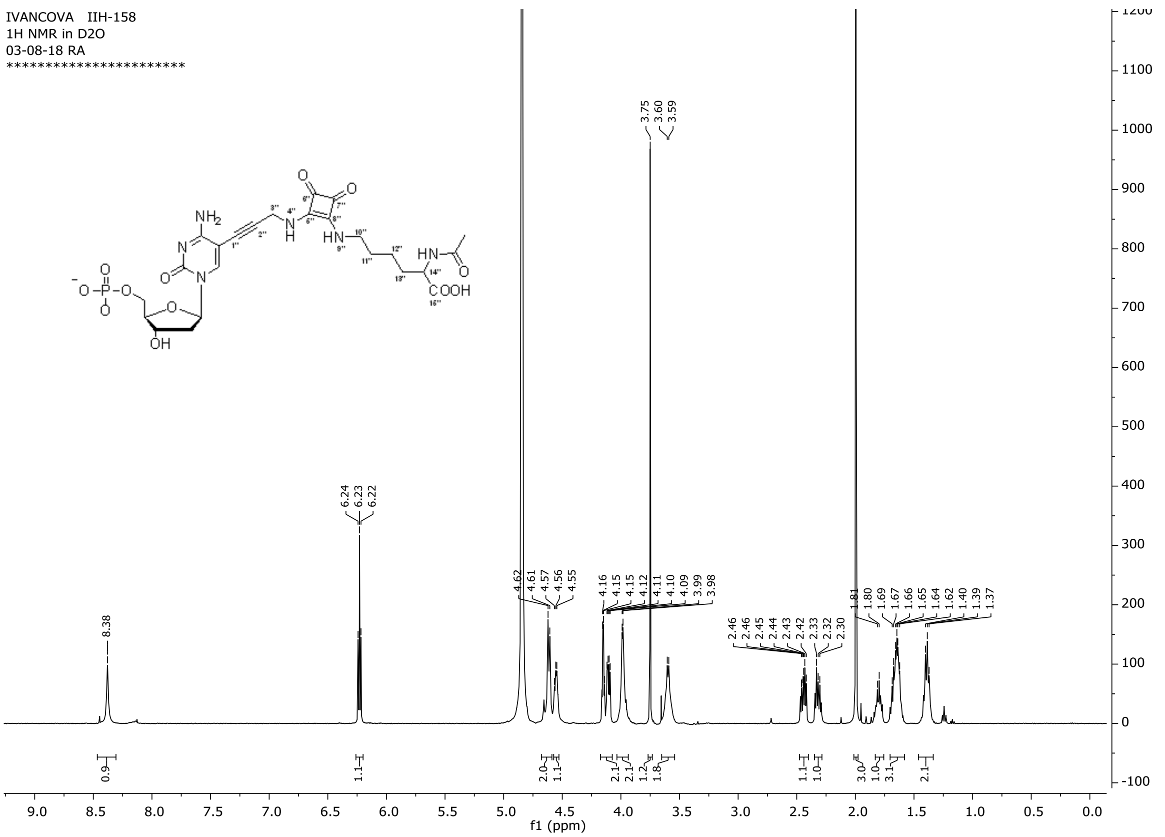
IVANCOVA IIH-158
1H NMR in D2O
03-08-18 RA
f1 (ppm)

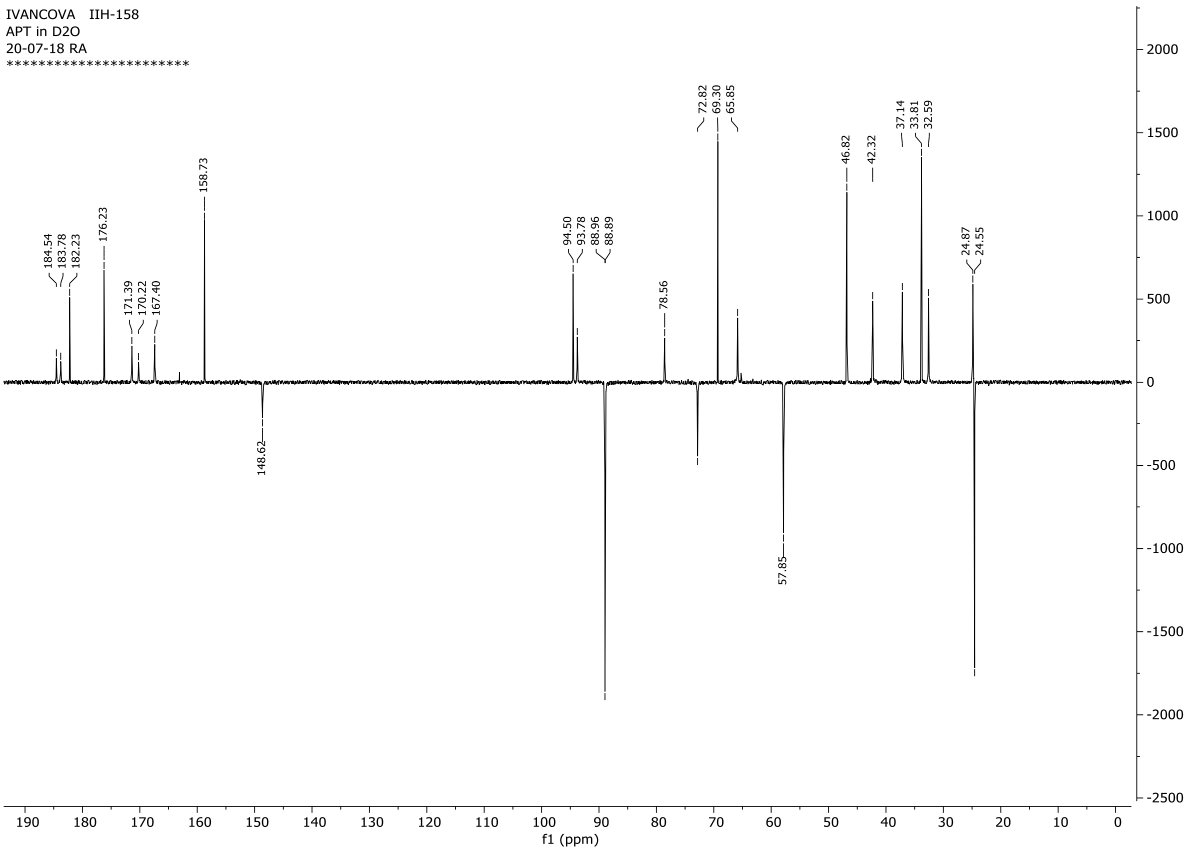
IVANCOVA IIH-158
APT in D2O
20-07-18 RA
f1 (ppm)

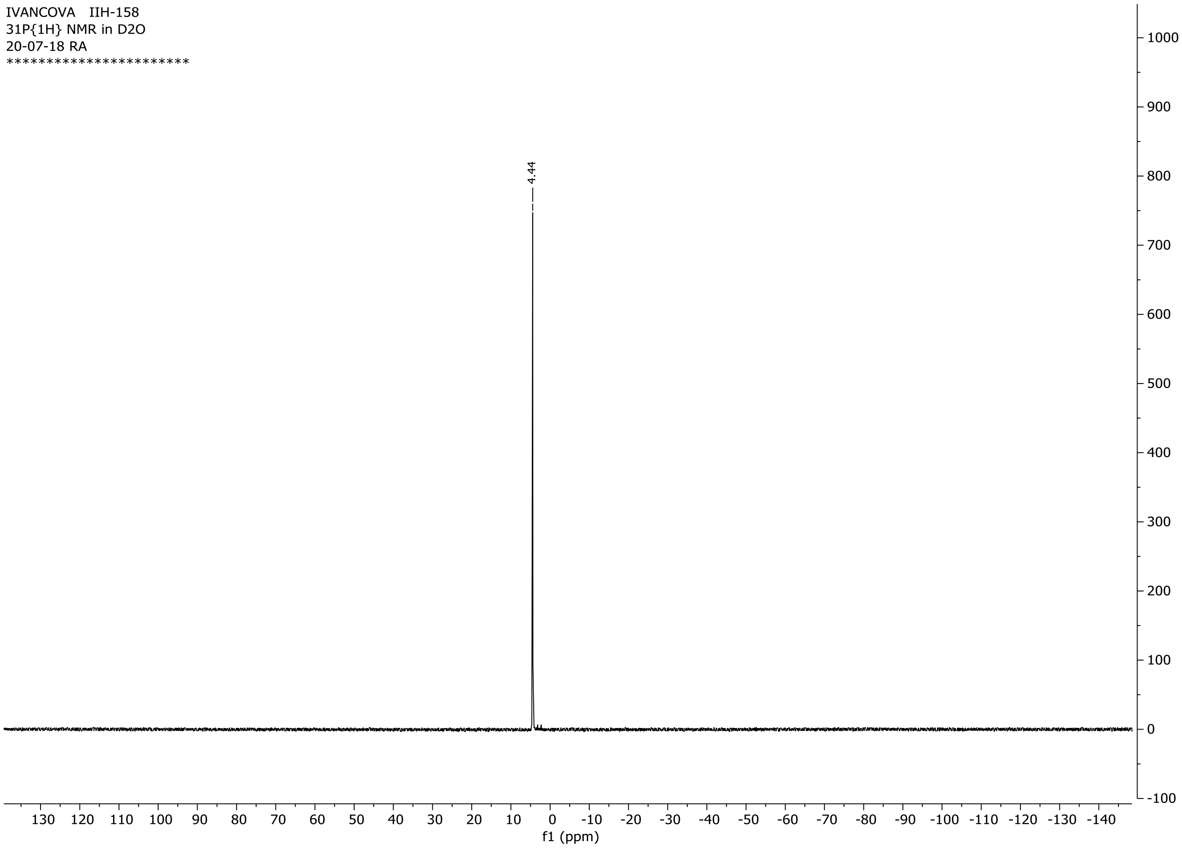
IVANCOVA IIH-158
31P{1H} NMR in D2O
20-07-18 RA
****
4.44
f1 (ppm)

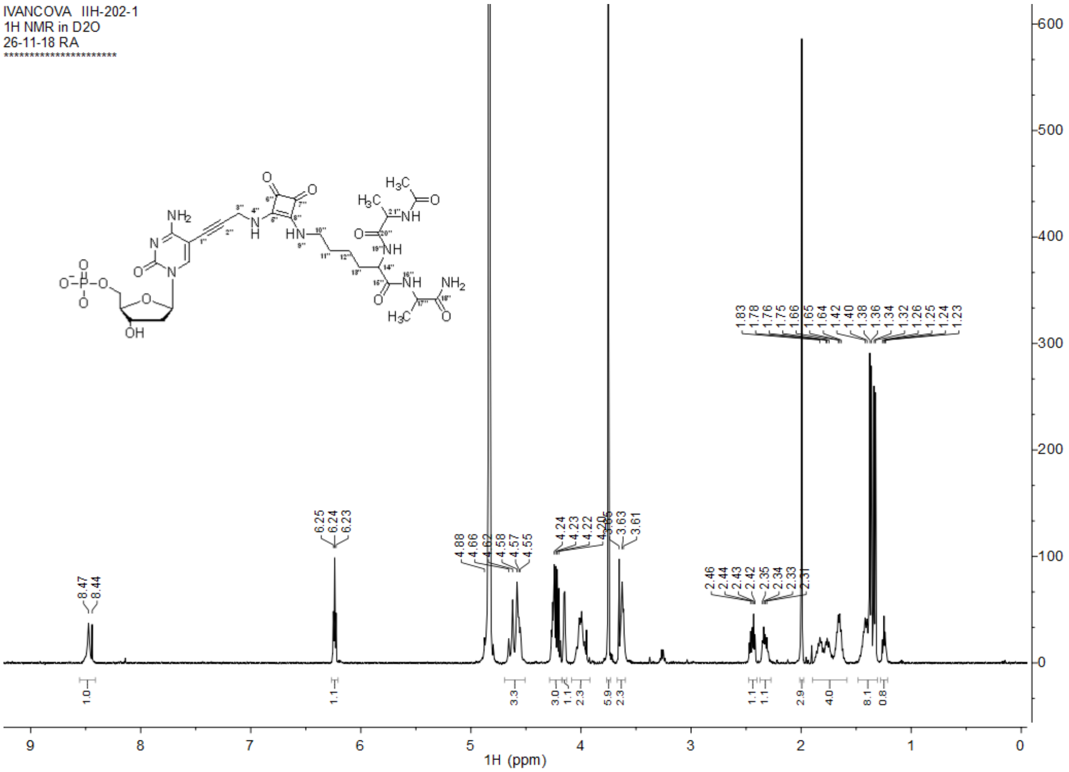
IVANCOVA IIH-202-1
1H NMR in D2O
26-11-18 RA
1H (ppm)

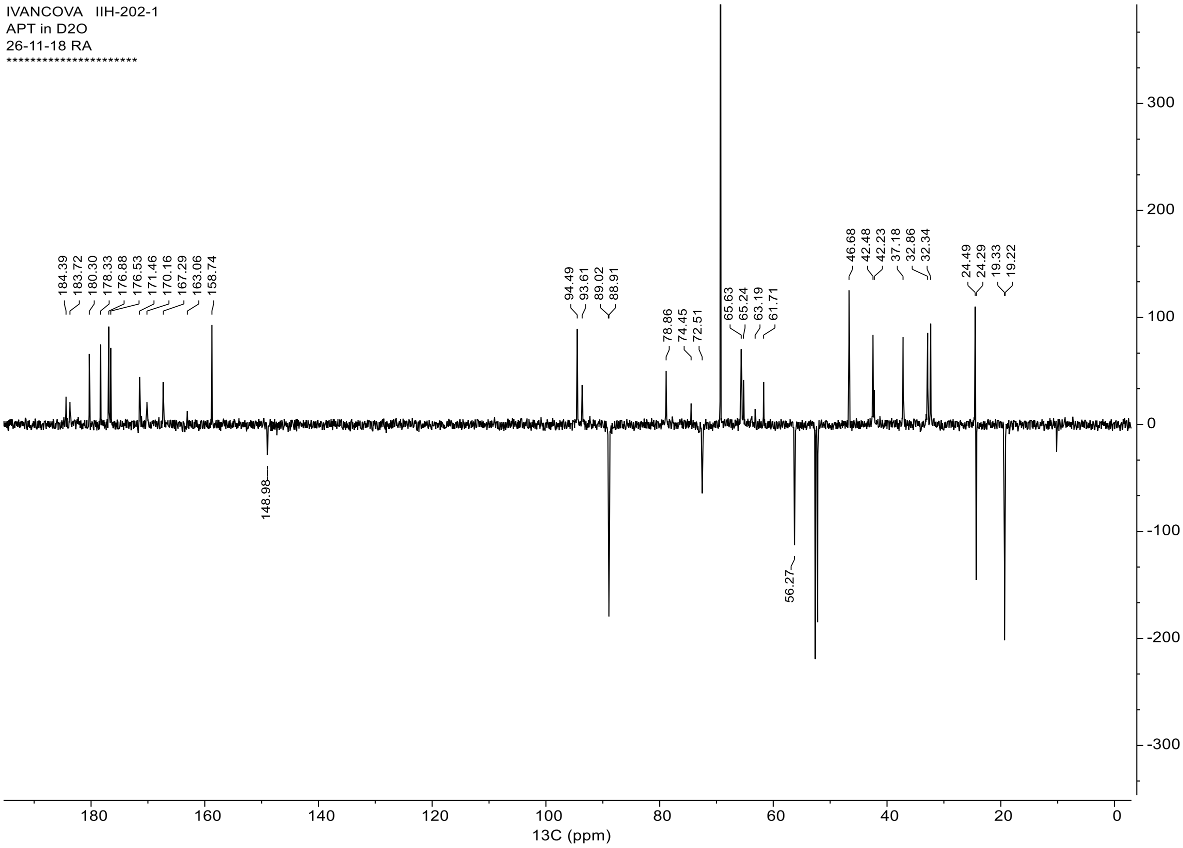
IVANCOVA IIH-202-1
APT in D2O
26-11-18 RA
13C (ppm)

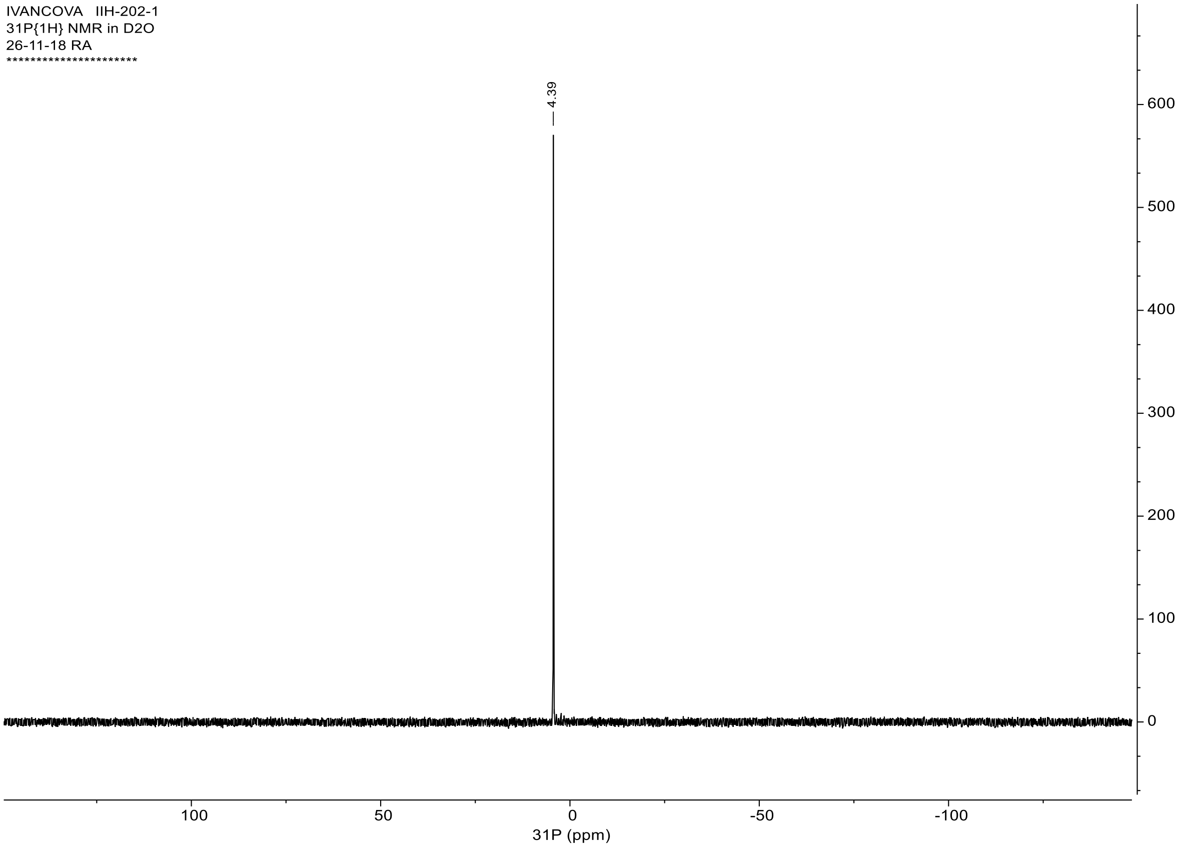
IVANCOVA IIH-202-1
31P{1H} NMR in D2O
26-11-18 RA
****
4.39
600
500
400
300
200
100
0
100
50
0
-50
-100
31P (ppm)

6. References

[1] A. S. K. Hashmi, T. Häffner, W. Yang, S. Pankajakshan, S. Schäfer, L. Schultes, F. Rominger, W. Frey, *Chem. Eur. J.* **2012**, *18*, 10480-10486.

[2] a) N. K. Garg, C. C. Woodroofe, C. J. Lacenere, S. R. Quake, B. M. Stoltz, *Chem. Commun.* **2005**, 4551-4553; b) S. Jäger, G. Rasched, H. Kornreich-Leshem, M. Engeser, O. Thum, M. Famulok, *J. Am. Chem. Soc.* **2005**, *127*, 15071-15082.

[3] A. G. Ferrige, M. J. Seddon, B. N. Green, S. A. Jarvis, J. Skilling, *Rapid Commun. Mass Spectrom.* **1992**, *6*, 707-711.
